# Diagnostic accuracy of circulating tumor DNA for detection of ALK rearrangement in lung cancer: A systematic review and meta-analysis of 14 studies

**DOI:** 10.1371/journal.pone.0330855

**Published:** 2025-08-25

**Authors:** Jiantong Sun, Lan Yang, Dan Liu, Hui Xue, Panwen Tian, Lei Li

**Affiliations:** 1 Department of Pulmonary and Critical Care Medicine, West China Hospital, Sichuan University, Chengdu, China; 2 Institute of Thoracic Oncology and Department of Thoracic Surgery, West China Hospital, Sichuan University, Chengdu, China; 3 Institute of Respiratory Health, Frontiers Science Center for Disease-related Molecular Network, West China Hospital, Sichuan University, Chengdu, China,; 4 West China Hospital, Sichuan University, Chengdu, China; 5 Lung Cancer Treatment Center, West China Hospital, Sichuan University, Chengdu, China; Seoul National University College of Pharmacy, KOREA, REPUBLIC OF

## Abstract

**Background:**

Circulating tumor DNA (ctDNA) is evolving into a promising non-invasive approach for the detection of ALK rearrangement. This meta-analysis was designed to determine the diagnostic value of ctDNA for ALK rearrangement in lung cancer patients.

**Methods:**

We performed a comprehensive publication search in Pubmed, Cochrane library, and Web of Science to identify the potentially relevant studies. Eligible studies were pooled to calculate the overall sensitivity, specificity, and diagnostic odds ratio (DOR). The area under the receiver operating-characteristic curve (AUC) was used to evaluate the overall diagnostic performance.

**Results:**

Thirteen eligible articles involving fourteen studies were identified in our meta-analysis, with a total of 1,138 participants. The pooled sensitivity, specificity, DOR, and AUC of ctDNA for ALK status detection were 0.61 (95% CI: 0.49, 0.72), 1.00 (95% CI: 0.98, 1.00), 188.19 (95% CI: 30.79, 1150.05) and 0.92, respectively. No publication bias was found among these studies (*P* = 0.42).

**Conclusion:**

Detecting ALK rearrangement in ctDNA demonstrates adequate diagnostic accuracy and could serve as a highly specific test in lung cancer patients.

## Introduction

Lung cancer is the most common fatal malignancy worldwide [1]. In 2022, nearly 2.5 million new cases of lung cancer were diagnosed worldwide, accounting for 12.4% of all cancer cases and making it the most frequently diagnosed cancer type. Moreover, lung cancer is the leading cause of cancer mortality globally, with an estimated 1.8 million deaths, representing 18.7% of all cancer deaths. Additionally, the number of new lung cancer cases is expected to increase significantly by 2050 [2]. Anaplastic lymphoma kinase (ALK) was found as a validated oncogenic driver in Non-Small Cell Lung Cancer (NSCLC) [3]. Studies have shown that ALK-positive NSCLC patients tend to be younger, and significant proportion of them have a never or light smoking history, in contrast to other NSCLC subsets. Histologically, adenocarcinoma is the common type, often with unique cellular subtypes such as signet ring cells. Interestingly, there is a relatively higher prevalence among men rather than women, which is different from some other genetic subtypes of NSCLC. Moreover, these patients usually lack other common oncogenic driver mutations like EGFR or KRAS mutations, highlighting the distinct molecular profile of this subgroup [3,4]. In recent years, ALK has drawn increasing attention as an effective therapeutic target of ALK-TKIs [5]. It has been demonstrated that crizotinib, the first-generation ALK-TKI, yielded a dramatic impact on ALK-positive NSCLC patients than traditional chemotherapy [6]. Other ALK inhibitors, including ceritinib and alectinib, have also shown an active clinical response. In 2011, ALK-TKIs were approved by the FDA as the first line therapy for ALK positive lung cancer patients [7]. Therefore, it is essential to determine the exact ALK status in lung cancer patients, in an effort to choose the optimal therapy and suitable surveillance plan.

Tissue-based assay has been regarded as the gold standard for ALK rearrangement testing. Immunohistochemistry (IHC) and fluorescence in-situ hybridization (FISH) are commonly used techniques for tissue samples. However, this testing system has shown some defects. Firstly, tissue or cytology specimens are sometimes insufficient or unobtainable, especially when disease progresses. Moreover, owing to the intra-tumoral and inter-metastatic heterogeneity, traditional tissue biopsy could not depict the global genomic landscape of the tumor [8]. Secondly, IHC and FISH testing can be subjective, with results varying among different examiners. The quality control analysis of IHC-VENTANA-D5F3 in the adenocarcinoma subgroup revealed an intra-hospital consistency rate of 98.2% and an inter-hospital consistency rate of 99.2%, but even these high consistency rates suggest some degree of variability [9]. Additionally, FISH may not be robust enough and can lead to false negative results. In a study on NRG1 fusion screening, although FISH was used to identify potential gene rearrangements, it had limitations and only a small number of cases were confirmed with NRG1 fusion through next-generation sequencing (NGS) [10].

Currently, circulating tumor DNA (ctDNA) has emerged as a promising minimally-invasive surrogate for tumor genotyping. EGFR-sensitizing and drug-resistant mutations analyses using ctDNA have been applied in clinical practice [11,12]. Alongside EGFR, inroads have also been made in the ctDNA-based assessment of ALK detection [13,14], allowing us to accurately detect genetic alteration profiling. Nevertheless, the diagnostic accuracy of ctDNA remains obscure. Based on such fact, we conducted this meta-analysis to investigate the diagnostic accuracy of ctDNA in detecting ALK status of lung cancer patients.

## Methods

### Data sources and search strategy

Potentially relevant studies were comprehensively searched on PubMed, Cochrane Library and Web of Science published up to February 11, 2024, without the beginning date and language limitations. The search was based on the combination of following terms: (“plasma”, “serum”, “cell free DNA”, or “circulating tumor DNA”), (“lung cancer” or “lung neoplasms”) and (“ALK” or “anaplastic lymphoma kinase”). In addition, we manually screened the references of eligible studies and relevant reviews to search for applicable records. In accordance with the Preferred Reporting Items for Systematic Reviews and Meta-analyses (PRISMA) guidelines [15], we conducted this analysis.

### Selection criteria

Researches were screened according to the following inclusion criteria: 1) all recruited patients should be diagnosed with lung cancer by cytology and/or histopathology confirmation, 2) the ALK status should be detected by ctDNA, using the results of tumor tissues as the reference standard, and 3) sufficient data were available to construct the diagnostic 2 × 2 table, 4) the studies must be original research articles published in peer-reviewed journals with clear and complete methodology and statistical analysis sections, 5) the study should have a clear definition of the diagnostic criteria for ALK-positive status, both in ctDNA and tumor tissues, and should report the concordance rate between the two detection methods, 6) the study should have been conducted within the last 10 years to ensure the relevance of the diagnostic techniques and clinical practices used. Records which met one of the following criteria were excluded: 1) duplicate articles, 2) reviews, case reports, guidelines, or protocols, 3) meeting abstracts published without full-text, 4) the ALK status were not detected by matched ctDNA and tumor tissues, 5)Researches that did not provide detailed information on the methods including the specific assays and platforms employed, 6) insufficient or unclear reporting of diagnostic accuracy metrics. Records retrieved were initially screened by titles and abstracts, and then the remaining articles were full-text cross reviewed by two reviewers. Any disagreements were discussed with the third reviewers to ensure consistency.

### Data extraction and quality assessment

Data extracted by two reviewers independently were as follows: name of the first author, publication year, ethnicity, sample size, age, percentage of female, smokers, and adenocarcina, TNM stage, specimen type and volume, whole blood volume, detection methods for ALK rearrangement in ctDNA, true-positive (TP), false-positive (FP), true-negative (TN), and false-negative (FN) values in each study. All discordances were solved by jointly discussing with the third author.

Quality assessment of these included studies was evaluated based on QUADAS-2 (the revised quality assessment of diagnostic accuracy studies 2) [16], and discrepancies between two independent investigators were resolved by mutual agreement. Each study was judged as “low”, “high”, or “unknown” in four categories, including patient selection, index test, reference standard, and flow and timing.

### Statistical analysis

We quantified the diagnostic accuracy by calculating the following pooled values and their 95% confidence intervals (CIs), including sensitivity, specificity, positive likelihood ratio (PLR), negative likelihood ratio (NLR) and diagnostic odds ratio (DOR). The forest plots and summary receiver operating-characteristic (SROC) curve were yielded to present the results visually, and then, and the area under the receiver operating-characteristic curve (AUC) was calculated.

Spearman correlations coefficient was applied to perform threshold effect analysis, with a *P*-value < 0.05 confirming the existence of threshold effect. The *P*-value of Cochrane’s Q test and the inconsistency index (*I*^2^) were measured to evaluate the statistical heterogeneity caused by non-threshold effect. When the P value≤0.05 by Q test, indicating the significant heterogeneity, a random-effect model was utilized. Otherwise, a fixed-effect model was used. Subgroup analyses were conducted to investigate the source of heterogeneity. Publication bias was evaluated by Deeks’ funnel plot asymmetry test. The stability of the pooled results was estimated using sensitivity analysis. Fagan nomogram was performed to evaluate the clinical utility of ALK detection in ctDNA.

Data analyses were performed with Stata version 14 (Stata Corp), Meta-DiSc version 1.4 and Review Manager version 5.3.

## Results

### Study selection and characteristics of included studies

**Fig 1:**
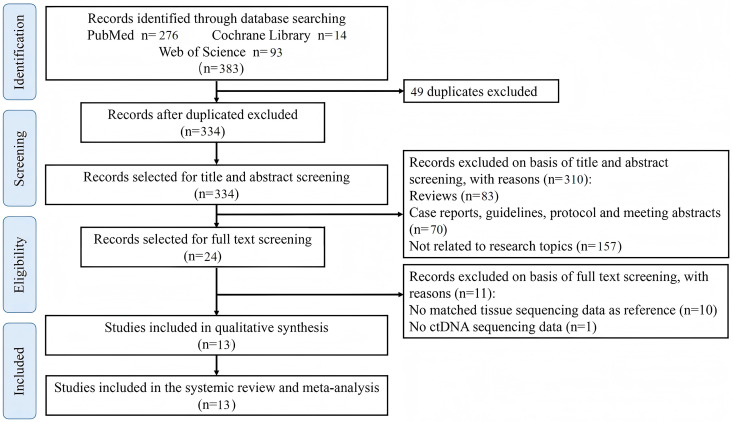
Retrieval flow chart of the included studies in this meta-analysis.

A total of 383 articles were retrieved, of which 49 duplicates were removed initially. After reviewing titles and abstracts, twenty-one potential articles remained. After full-text reading, 13 selected articles were available for analysis [17–29], published during 2017–2023. Notably, the study reported by RJA Nilsson [17] detected ALK rearrangement both in plasma and platelets. We analyzed these data as two independently studies. Therefore, 14 eligible studies involving 1,138 participants were incorporated into meta-analysis (Fig 1).

Among all the included researches, five were conducted in the United States [19,22,23,25,27], six in China [18,20,21,26,28,29], one in Germany [24], and a multi-center research was conducted in Netherlands, Spain, and the USA [25]. The involved 478 lung cancer patients were diagnosed at advanced stage (III-IV) in most cases, with the sample size between 6 and 103. The volume of whole blood samples varied from 6 to 10 mL. ALK rearrangement were detected in serum samples [25], platelet [25] and plasma samples [15–22,24–27], comparing with the results in tissue samples as the gold standard reference. The volume of serum/plasma specimen ranged from 200 μL to 5mL. The detection method of ctDNA contained NGS and RT-RCR. Further details of all selected studies are summarized in Table 1.

**Table 1:** Baseline characteristics of the included studies.

Author	Year	Ethnicity	Size	Age, Median (range)	Male (%)	Smoker (%)	Clinical Stage	Blood Sample	Specimen volume	Whole Blood Volume	Method	No.of TP	No.of FP	No.of FN	No.of TN
N Guibert	2018	American	9	NA	NA	NA	IIIB/IV	Plasma	1-2 mL	NA	Amplicon-based plasma NGS	6	0	1	2
Z Wang	2017	Chinese	103	64 (21-87)	47	32	III/IV	Plasma	2 mL	10 mL	Single-molecule counting cSMART assay (RT-PCR)	3	0	3	97
SH Cui	2017	Chinese	39	55 (31-73)	69	36	IB/IIIA/IIIB/IV	Plasma	4-5 ml	10 mL	Capture-based NGS	13	0	11	15
Y Yao	2017	Chinese	39	62 (28-78)	49	26	IIIA/IIIB/IV	Plasma	5 mL	NA	Capture-based targeted sequencing (NGS)	3	0	1	35
H Mellert	2017	American	24	NA	NA	NA	NA	Plasma	4-5 ml	10 mL	digital PCR (RT-PCR)	10	0	5	9
CP Paweletz	2016	American	49	57 (NA)	39	NA	NA	Plasma	NA	10 mL	Bias-Corrected Targeted NGS	2	0	1	46
S Dietz	2016	German	6	NA	50	83	IIIA/IIIB	Serum	200 μL	NA	WES (NGS)	1	0	0	5
JC Thompson	2016	American	50	64 (34-85)	34	50	II/III/IV	Plasma	NA	10 mL	NGS	1	1	0	48
RJA Nilsson 1	2016	Multi-center	32	NA	NA	NA	NA	Plasma	NA	6 mL	RT-PCR	3	0	11	18
RJA Nilsson 2	2016	Multi-center	67	NA	NA	NA	NA	Platelets	NA	6 mL	RT-PCR	22	0	12	33
Y Wang	2016	Chinese	60	48 (30-66)	42	42	IIIB/IV	Plasma	4-5 ml	10 mL	Capture-based targeted sequencing (NGS)	19	0	5	36
Natasha B. Leighl *	2019	American	282	69 (26-100)	45.7	21.7	IIIB/IV	Plasma	NA	NA	Guardant 360 (NGS)	6	0	2	207
H Yang	2022	Chinese	22	54 (38-74)	50	NA	NA	Plasma	NA	NA	NGS	1	0	1	20
Jianjiang Xie	2023	Chinese	423	63 (30-88)	53	53	IA/II/III/IV	Plasma	NA	NA	NGS	3	5	6	409

Abbreviations: AC, Adenocarcinoma; NGS, Next-generation sequencing; WSE, Whole-Exome Sequencing; AUC, Area under summary receiver operating characteristic curve; cSMART, circulating single-molecule amplification and resequencing technology; RT-PCR, Real-time reverse transcription-polymerase chain reaction; TP, true-positive; FP, false-positive; FN, false-negative; TN, true-negative; NA, not available.

### Quality assessment and publication bias

**Fig 2:**
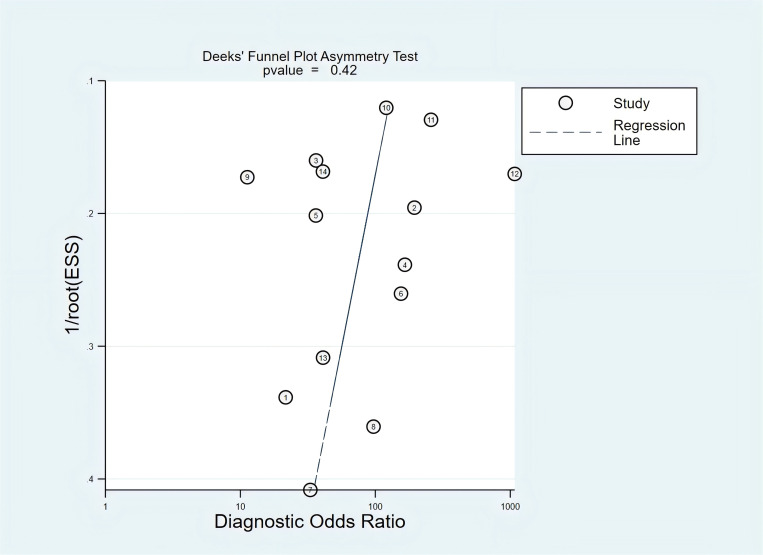
The Deek’s funnel plot asymmetry test.

The methodological quality assessment is provided in S1 Fig. The risk of bias of selected studies of included studies was moderate-high, and applicability was high for all studies. By using the Deeks’ funnel plot asymmetry test, no significant differences were detected in this meta-analysis (P-value = 0.42, Fig 2).

### Heterogeneity and sensitivity analysis

No significant threshold effect was identified (r = 0.424, P = 0.194). Sensitivity analysis was conducted, and no significant results were found (S2 Fig).

### Diagnostic accuracy of ctDNA

For all studies, the pooled sensitivity, specificity, PLR, and NLR of ctDNA for detecting ALK rearrangement was 0.61 (95% CI: 0.49, 0.72), 1.00 (95% CI: 0.98, 1.00), 23.86 (95% CI: 9.82, 58.02), and 0.39 (95% CI: 0.28, 0.53), respectively. The DOR and AUC were 188.19 (95% CI: 30.79, 1150.05) and 0.92 (Table 2, S3 Fig), indicating the good diagnostic accuracy of ctDNA.

**Table 2:** Pooled results of this meta-analyses.

	No. of studies	SEN (95% CI)	P	SPE (95% CI)	P	PLR (95% CI)	P	NLR (95% CI)	P	DOR (95% CI)	P	AUC
**Overall**	14	0.61 [0.49-0.72]	0.07	1.00 [0.98-1.00]	0.49	188.19 [30.79-1150.05]	<0.01	0.39 [0.28-0.53]	<0.01	485.73 [72.45-3256.52]	<0.01	0.98
**Ethnicity**												
Chinese	6	0.61 [0.48-0.72]	0.18	0.99 [0.98-1.00]	0.55	33.32 [13.60-81.63]	0.94	0.46 [0.32-0.65]	0.18	71.00 [24.27-207.70]	0.82	0.98
Non-Asia	8	0.61 [0.50-0.72]	0.05	1.00 [0.98-1.00]	0.77	23.06 [9.39-56.62]	0.5	0.40 [0.23-0.67]	0	73.25 [22.82-235.05]	0.59	0.89
**TNM Stages**												
I-IV	3	0.50 [0.32-0.68]	0.28	0.99 [0.97-1.00]	0.74	25.30 [9.63-66.44]	0.87	0.55 [0.40-0.76]	0.45	44.95 [11.89-169.99]	0.9	0.08
III-IV	6	0.76 [0.62-0.87]	0.7	1.00 [0.99-1.00]	1	37.07[10.61-129.51]	0.29	0.31 [0.20-0.47]	0.75	167.95 [43.05-655.27]	0.65	0.9
**Smoker**												
< 50%	3	0.71 [0.42-0.92]	0.26	1.00 [0.97-1.00]	1	15.00[2.50-90.01]	0.27	0.42 [0.22-0.79]	0.61	61.14 [7.78-480.30]	0.61	0.89
≥ 50%	5	0.68 [0.56-0.78]	0.44	1.00 [0.99-1.00]	1	44.81 [12.99-154.57]	0.52	0.36 [0.26-0.50]	0.35	136.88 [34.68-540.30]	0.45	0.66
**Specimen volume**												
< 3mL	5	0.67 [0.54-0.78]	0.34	1.00 [0.99-1.00]	1	67.38 [19.29-235.28]	0.65	0.38 [0.26-0.54]	0.31	193.54 [48.49-772.41]	0.59	0.87
≥ 3mL	2	1.00 [0.16-1.00]	1	0.98 [0.90-1.00]	0.66	18.61 [4.19-82.59]	0.48	0.27 [0.05-1.45]	0.97	62.42 [3.95-985.87]	0.69	NA
**Detection approaches of ctDNA**												
NGS	10	0.66 [0.55-0.76]	0.26	0.99 [0.98-1.00]	0.61	28.07 [13.79-57.17]	0.73	0.42 [0.31-0.57]	0.3	79.24 [31.38-200.11]	0.8	0.93
RT-PCR	4	0.55 [0.43-0.67]	0.03	1.00 [0.98-1.00]	1	26.45 [6.50-107.59]	0.63	0.49 [0.29-0.84]	0	55.87 [12.33-253.07]	0.57	0.92

Abbreviations: SEN, Sensitivity; SPE, Specificity; PLR, Positive likelihood ratio; NLR, Negative likelihood ratio; DOR, Diagnostic odds ratio; AUC, Area under summary receiver operating characteristic curve; NGS, Next-generation sequencing; RT-PCR, Real-time reverse transcription-polymerase chain reaction.

The stratified analysis results of ctDNA were listed in Table 2, according to ethnicity, TNM stages, the percentage of smokers, specimen volume, and the detection approaches of ctDNA. We observed that the sensitivity was higher in patients with advanced TNM stages (III-IV) (0.76, 95% CI: 0.62–0.87) compared to those with stages I-IV (0.50, 95% CI: 0.32–0.68), which may be attributed to higher tumor burden and ctDNA release in advanced disease. The percentage of smokers did not significantly alter the sensitivity (≥50% smokers: 0.68 vs. < 50%: 0.71). However, the specimen volume showed a trend toward higher sensitivity with volumes ≥3 mL (1.00) compared to <3 mL (0.67), though the number of studies in the ≥ 3 mL group was limited (n = 2). These factors (TNM stage, specimen volume) were identified as potential sources of heterogeneity.

### Clinical utility assessment

**Fig 3:**
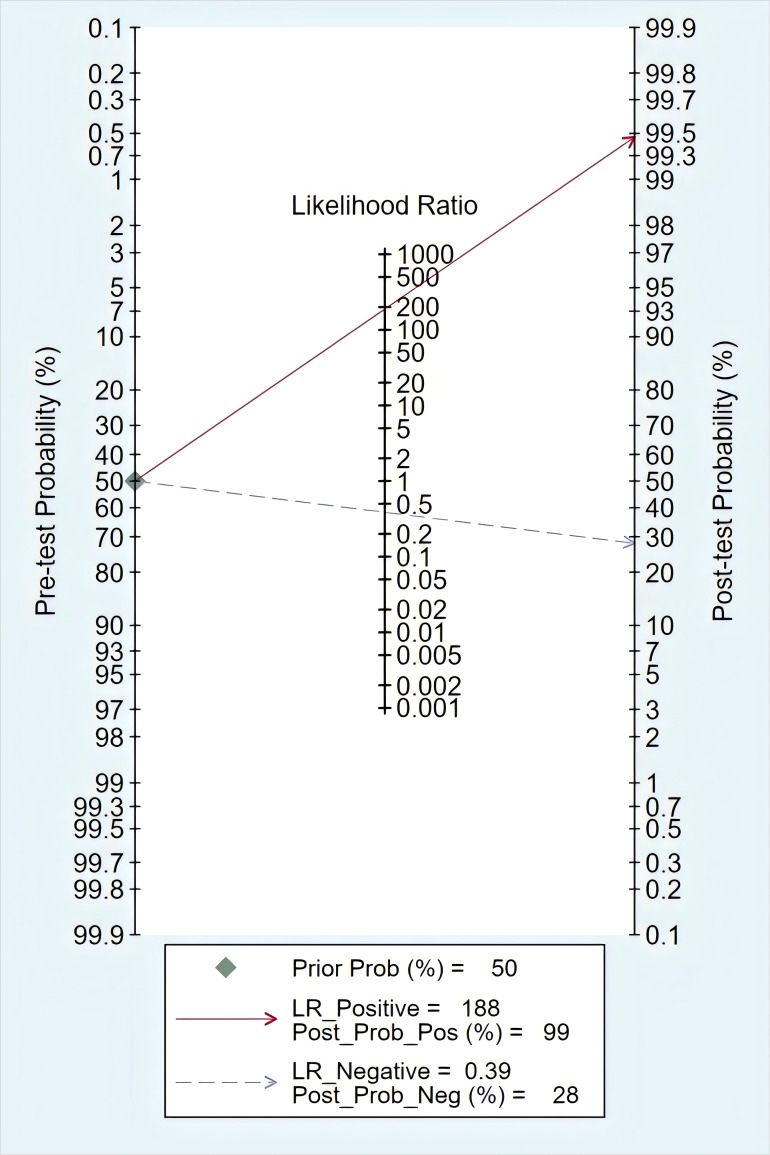
Fagan nomogram to assess the clinical utility of ctDNA in ALK rearrangement detection.

The Fagan nomogram existed a dramatic improvement of post-test probability (Fig 3). When 20% was set as the pre-test probability for ALK status detection, the post-test probability of ctDNA-positive surged to 99%, with the post-test probability of ctDNA-negative dropping to 8%.

## Discussion

ALK-TKI therapy has significantly improved the prognosis of ALK-positive lung cancer patients, making ALK test a routine examination in most NSCLC cases. Traditional detecting methods have some prominent limitations, including unavailability of adequate tumor tissue samples and infeasibility of a real-time surveillance. Blood samples provided a much easier and more widely available way for detection, and was considered as ideal surrogate. However, no further study on the theme of diagnostic accuracy of ctDNA for ALK rearrangement detection has been reported.

In this meta-analysis, ctDNA-based ALK detection yielded a significantly high specificity of 1.00, and a moderate sensitivity of 0.61. Similar results have been verified in F Passiglia’s study [30], which emphasized that EGFR mutation analyses in ctDNA was reliable for NSCLC, with a highly specificity (0.80) but a moderate sensitivity (0.67). In most cases, the PLR value greater than 10 and the NLR value less than 0.1 served as useful indicators to rule in and rule out diagnoses, respectively [31]. The pooled PLR for ctDNA was 23.86, indicating that the positive result of ctDNA-based ALK testing could be reliable and of great practical value in avoiding unnecessary tissue biopsy. But for the NLR of 0.41, false negative cases were possible if detecting ctDNA alone. Therefore, further tissue analysis was critical for ctDNA-ALK negative patients to avoid false negative cases.

The Fagan nomogram analysis quantitatively confirmed these clinical implications: a positive ctDNA result (99% post-test probability) provides near-definitive evidence for ALK rearrangement, supporting immediate targeted therapy initiation without tissue confirmation in appropriate clinical settings. Conversely, the 8% residual probability after negative ctDNA necessitates mandatory tissue biopsy when clinical suspicion persists, establishing ctDNA as a high-confidence rule-in test but not a rule-out modality.

The NLR higher than 0.1 may in part be a consequence of the moderate sensitivity. It was found that TNM stage, percentage of smokers and the specimen volume were potential causes of the heterogeneity, which might result in lower overall sensitivity. It was found that the use of ctDNA detection technology for detecting lung cancers of different histological classifications yields varying sensitivities. However, due to the lack of available histological classification information in existing articles, we were unable to conduct subgroup analyses on the included articles according to histological classifications and further analyze the specific causes of the moderate sensitivity. In addition, the DOR and AUC were found to be 188.19 and 0.92, indicating the competitive diagnostic performance of ctDNA in detecting ALK status. From the results yielded in our pooled results, it was demonstrated that ctDNA-based ALK rearrangement analysis could be used as a reliable screening test before a biopsy.

Currently, ctDNA could be separated from both serum and plasma. Though several studies reported a significantly higher ctDNA concentration in serum, plasma could be a better source of ctDNA, without the contamination of genomic DNA from white blood cells [32]. In our meta-analysis, most original studies extracted ctDNA from plasma, while only two studies used serum [24] and platelets [17].As is reported by RJA Nilsson [17], platelets showed excellent sensitivity than plasma in ALK testing (65% vs 21%), suggesting platelets as better sources. It might be a valuable topic for further studies to explore the feasibility of detecting ALK status in platelets samples.

Different techniques have been used to determine ALK rearrangement, including IHC, FISH, reverse transcription polymerase chain reaction (RT-PCR) and next-generation sequencing (NGS) [33]. We noticed that NGS was the most frequently used method for ctDNA, with a higher sensitivity (0.70 vs. 0.55) than RT-PCR. Similar to our results, Ignor Letovanec noted that tissue-based NGS achieved a higher sensitivity and lower specificity than tissue-based RT-PCR assays [13]. Notably, the NGS-based diagnostic assay exhibited several advantages over traditional methods. Firstly, it could detect novel and complex ALK fusions. Secondly, it is more reliable to pick out the positive patients who may benefit from Crizotinib, since NGS-positive patients had significantly longer PFS compared with NGS-negative cases [14]. Therefore, despite its high cost and the complex detection procedure, NGS-based assays are achieving broad applications in detecting ALK rearrangements of lung cancer patients.

In view of TNM stage, we found that the sensitivity, specificity, and DOR were considerably higher for patients with advanced stage (stages III–IV). Analogous results were conducted in EGFR, showing that sensitivities in patients with stage IIIB-IV and stage IA–IIIA were 72.7%, and 22.2%, respectively [34]. Clinical tumor stage could affect the sensitivities [21]. The possible reason might be different abundance of ctDNA in different stages [35,36], with advanced NSCLC released more ctDNA fragments, resulting in relatively higher ctDNA abundance and concentration. In general, ctDNA-based ALK detection may perform better in patients with advanced lung cancer, with high clinical utility.

This is the first meta-analysis focusing on the diagnostic value of ALK rearrangement by ctDNA in lung cancer. However, several limitations should be taken into account. First of all, only 13 articles were integrated after strict selection, which might influence the statistical power to reach a definitive conclusion. In addition, although no language limitation was applied, only studies published in English and Chinese were included, which might result in publication bias, though the Deeks regression test showed no significant publication bias in the present analysis. Furthermore, the relationship between blood collection, treatment, and outcome of patients may result in bias. Unfortunately, it could not be analyzed due to the insufficient details in limited studies. The moderate pooled sensitivity of 0.61 (95% CI: 0.49–0.72) reflects fundamental biological and technical constraints: the lower shedding of fusion transcripts compared to point mutations, stage-dependent detect ability limitations (particularly in early-stage disease), and vulnerability to pre-analytical variables like blood collection volume and processing delays. Further studies are required to investigate these issues and validate our conclusions.

As a non-invasive biomarker, ctDNA offers several advantages over traditional tissue biopsies, including reduced procedural risks, faster turnaround times, and the ability to provide a more comprehensive representation of tumor heterogeneity [37]. This application is especially valuable when tissue samples are insufficient or unavailable, as seen in up to 30% of advanced NSCLC cases [38]. However, the clinical utility of ctDNA is not without limitations. One major concern is the potential for false-negative results, particularly in patients with low tumor burden or when ctDNA levels fall below the detection threshold of current assays. This can lead to missed diagnoses or inappropriate treatment decisions [30,39]. Especially when the tumor burden is low, detecting gene fusions and alterations is more difficult in ctDNA than in tissues, which could be an vital reason for its relatively low sensitivity [40]. Conversely, false-positive results may arise from the detection of genetic alterations originating from non-cancerous sources, such as clonal hematopoiesis of indeterminate potential (CHIP), which can complicate the interpretation of ctDNA findings [41]. The prevalence of CHIP increases with age and assay sensitivity, potentially leading to misinterpretation of ctDNA mutations [42]. These limitations highlight the need for further optimization of ctDNA assays, including improved sensitivity and specificity, as well as standardized pre-analytical and analytical protocols. Additionally, combining ctDNA analysis with other biomarkers or imaging modalities may enhance diagnostic accuracy and reduce the risk of false results [43]. Future research should focus on addressing these challenges to ensure that ctDNA-based tests can be reliably integrated into clinical practice, ultimately enhancing the precision and effectiveness of lung cancer management [44].

While the primary objective of this study was not to evaluate the cost-effectiveness of ctDNA analysis, the cost-effectiveness of ctDNA analysis remains a significant consideration. ctDNA detection techniques, such as next-generation sequencing and digital PCR, are often expensive and require specialized equipment and expertise [45]. Despite these costs, the potential benefits of ctDNA analysis, including early detection of treatment resistance and personalized treatment strategies, may outweigh the financial burden. For example, in a study evaluating the cost-effectiveness of ctDNA-guided treatment decisions in a different cancer type, the incremental cost-effectiveness ratio was found to be €65,400.86 per quality-adjusted life-year (QALY) gained, indicating that ctDNA analysis can be cost-effective in certain clinical scenarios [46]. While the cost of ctDNA analysis is an important factor, the clinical benefits associated with early detection of ALK rearrangements and personalized treatment approaches suggest that ctDNA analysis could be a valuable tool in the management of ALK-rearranged NSCLC. Future research should focus on optimizing the cost-effectiveness of ctDNA analysis through improved detection techniques and standardized protocols.

In conclusion, the present meta-analysis demonstrated the feasibility and utility of ctDNA in detecting ALK rearrangements for lung cancer patients, particularly for whom matched tissue is not available. Given the high diagnostic accuracy and specificity, ctDNA could serve as a promising substitute of tumor tissues in lung cancer patients.

## Supporting information

S1 Appendix PRISMA 2009 checklist. (PDF)

S1 Table Included articles & Excluded articles with reasons. (PDF)

S2 Table Characteristics of eligible studies. (S2 Table.PDF)

S3 Table QUADAS-2 tool assessment results. (S3 Table.PDF)

S1 Fig Quality assessment for included studies in this meta-analysis. (TIF)

S2 Fig Sensitivity analysis of these eligible studies. (TIF)

S3 Fig Forest plots of the analysis results of ctDNA: (A) sensitivity, (B) specificity, (C) positive likelihood ratio, (D) negative likelihood ratio, (E) diagnostic odds ratio, and (F) SROC curve. (TIF)
