# Supplementary material for: Diagnostic accuracy of circulating tumor DNA for detection of ALK rearrangement in lung cancer: A systematic review and meta-analysis of 14 studies
Source: PLoS One. 2025 Aug 25;20(8):e0330855. doi: 10.1371/journal.pone.0330855 (PMC12377591; doi:10.1371/journal.pone.0330855)
Supplement: S1 Table — (PDF) [file pone.0330855.s002.pdf]

Supplementary Table 1. Excluded articles with reasons & Included articles.

Excluded articles							
	Title	Exclusion reason	Authors	First Author	Journal	Publication Year	DOI/URL
1	Circulating lung cancer biomarkers: From translational research to clinical practice	Reviews	Qian X, Meng QH.	Qian X	Tumour Biol	2024	10.3233/TUB-230012
2	Prospective observational study to explore genes and proteins predicting efficacy and safety of brigatinib for ALK-gene rearranged non-small-cell lung cancer: study protocol for ABRAID study (WJOG11919L)	Case reports, guidelines, protocol and meeting abstracts	Ozawa Y, Koh Y, Hase T, Chibana K, Kaira K, Okishio K, Ichihara E, Murakami S, Shimokawa M, Yamamoto N.	Ozawa Y	Ther Adv Med Oncol	2024	10.1177/17588359231225046
3	EML4-ALK fusion protein in Lung cancer cells enhances venous thrombogenicity through the pERK1/2-AP-1-tissue factor axis	Data not related or missing	Su Y, Yi J, Zhang Y, Leng D, Huang X, Shi X, Zhang Y.	Su Y	J Thromb Thrombolysis	2024	10.1007/s11239-023-02916-5
4	Lung adenocarcinoma with brain metastasis detected dual fusion of LOC399815-ALK and ALK-EML4 in combined treatment of Alectinib and CyberKnife: A case report	Case reports, guidelines, protocol and meeting abstracts	Li Y, Lu S, Yao P, Huang W, Huang Y, Zhou Y, Yuan Y, Cheng S, Wu F.	Li Y	Medicine (Baltimore)	2024	10.1097/MD.0000000000036992
5	Clinical Relevance of High Plasma Trough Levels of the Kinase Inhibitors Crizotinib, Alectinib, Osimertinib, Dabrafenib, and Trametinib in NSCLC Patients	Data not related or missing	Lin L, Barkman HJ, Smit EF, de Langen AJ, Steeghs N, Beijnen JH, Huitema ADR.	Lin L	Ther Drug Monit	2024	10.1097/FTD.0000000000001120
6	Cerebrospinal fluid ctDNA testing shows an advantage over plasma ctDNA testing in advanced non-small cell lung cancer patients with brain metastases	Data not related or missing	Liu X, Mei F, Fang M, Jia Y, Zhou Y, Li C, Tian P, Lu C, Li G.	Liu X	Front Oncol	2024	10.3389/fonc.2023.1322635
7	Evaluation of the drug-drug interaction potential of brigatinib using a physiologically-based pharmacokinetic modeling approach	Data not related or missing	Hanley MJ, Yeo KR, Tugnait M, Iwasaki S, Narasimhan N, Zhang P, Venkatakrishnan K, Gupta N.	Hanley MJ	CPT Pharmacometrics Syst Pharmacol	2024	10.1002/psp4.13106
8	Strategies to overcome resistance to ALK inhibitors in non-small cell lung cancer: a narrative review	Reviews	Desai A, Lovly CM.	Desai A	Transl Lung Cancer Res	2023	10.21037/tlcr-22-708
9	Comparative safety of anaplastic lymphoma kinase tyrosine kinase inhibitors in advanced anaplastic lymphoma kinase-mutated non-small cell lung cancer: Systematic review and network meta-analysis	Reviews	Luo Y, Zhang Z, Guo X, Tang X, Li S, Gong G, Gao S, Zhang Y, Lin S.	Luo Y	Lung Cancer	2023	10.1016/j.lungcan.2023.107319
10	Efficacy of Lorlatinib in Treatment-Naive Patients With ALK-Positive Advanced NSCLC in Relation to EML4::ALK Variant Type and ALK With or Without TP53 Mutations	Data not related or missing	Bearz A, Martini JF, Jassem J, Kim SW, Chang GC, Shaw AT, Shepard DA, Dall'O' E, Polli A, Thurm H, Zalcman G, Garcia Campelo MR, Penkov K, Hayashi H, Solomon BJ.	Bearz A	J Thorac Oncol	2023	10.1016/j.jtho.2023.07.023
11	Early-stage anaplastic lymphoma kinase (ALK)-positive lung cancer: a narrative review	Reviews	Chen MF, Chaft JE.	Chen MF	Transl Lung Cancer Res	2023	10.21037/tlcr-22-631
12	Molecular and Genetic Advances in Small Cell Lung Cancer Landscape: From Homogeneity to Diversity	Reviews	Zullo L, Dall'Olio FG, Rossi G, Dellepiane C, Barletta G, Bennicelli E, Ingaliso M, Tagliamento M, Genova C.	Zullo L	Int J Mol Sci	2023	10.3390/ijms25010224
13	A Review of Biomarkers and Their Clinical Impact in Resected Early-Stage Non-Small-Cell Lung Cancer	Reviews	Cao W, Tang Q, Zeng J, Jin X, Zu L, Xu S.	Cao W	Cancers (Basel)	2023	10.3390/cancers15184561

14	ALK-positive lung cancer: a moving target	Reviews	Jaime L Schneider, Jessica J Lin, Alice T Shaw	Jaime L Schneider	Nat Cancer	2023	10.1038/s43018-023-00515-0
15	Integrated circulating tumour DNA and cytokine analysis for therapy monitoring of ALK-rearranged lung adenocarcinoma	Data not related or missing	Angeles AK, Janke F, Daum AK, Reck M, Schneider MA, Thomas M, Christopoulos P, Sültmann H.	Angeles AK	Br J Cancer	2023	10.1038/s41416-023-02284-0
16	The Implication of Liquid Biopsy in the Non-small Cell Lung Cancer: Potential and Expectation	Reviews	Ren J, Liu R.	Ren J	Methods Mol Biol	2023	10.1007/978-1-0716-3346-5_10
17	Molecular heterogeneity and co-altered genes in MET-amplified ALK-positive lung cancer: Implications for MET targeted therapy	Data not related or missing	Dagogo-Jack I, Kiedrowski LA, Lennerz JK.	Dagogo-Jack I	Lung Cancer	2023	10.1016/j.lungcan.2023.107383
18	Non-Small Cell Lung Cancer: Targetable Variants in Concurrent Tissue and Liquid Biopsy Testing in a North Indian Cohort	Case reports, guidelines, protocol and meeting abstracts	Paturu R, Lingaiah R, Kumari N, Singh S, Krishnani N, Srivastava S, Siddiqui SH, Nath A.	Paturu R	Asian Pac J Cancer Prev	2023	10.31557/APJCP.2023.24.10.3467
19	Randomized, open-label phase II study of brigatinib and carboplatin plus pemetrexed and brigatinib alone for chemotherapy-naive patients with ALK-rearranged non-squamous non-small cell lung cancer: treatment rationale and protocol design of the B-DASH study (WJOG 14720 L)	Case reports, guidelines, protocol and meeting abstracts	Wakuda K, Kenmotsu H, Sato Y, Nakamura A, Akamatsu H, Tachihara M, Miura S, Yokoyama T, Mori K, Nakagawa K, Yamamoto N.	Wakuda K	BMC Cancer	2023	10.1186/s12885-023-11417-w
20	Analysis of Serious Weight Gain in Patients Using Alectinib for ALK-Positive Lung Cancer	Data not related or missing	de Leeuw SP, Pruis MA, Sikkema BJ, Mohseni M, Veerman GDM, Paats MS, Dumoulin DW, Smit EF, Schols AMWJ, Mathijssen RHJ, van Rossum EFC, Dingemans AC.	de Leeuw SP	J Thorac Oncol	2023	10.1016/j.jtho.2023.03.020
21	Efficacy and Tolerability of ALK/MET Combinations in Patients With ALK-Rearranged Lung Cancer With Acquired MET Amplification: A Retrospective Analysis	Data not related or missing	Dagogo-Jack I, Kiedrowski LA, Heist RS, Lin JJ, Meador CB, Krueger EA, Do A, Peterson J, Sequist LV, Gainor JF, Lennerz JK, Digumarthy SR.	Dagogo-Jack I	JTO Clin Res Rep	2023	10.1016/j.jtocrr.2023.100534
22	Brigatinib pharmacokinetics in patients with chronic hepatic impairment	Reviews	Hanley MJ, Kerstein D, Tugnait M, Narasimhan N, Marbury TC, Venkatakrishnan K, Gupta N.	Hanley MJ	Invest New Drugs	2023	10.1007/s10637-023-01339-6
23	Resistance to immune checkpoint inhibitors in advanced lung cancer: Clinical characteristics, potential prognostic factors and next strategy	Reviews	Zhou J, Lu X, Zhu H, Ding N, Zhang Y, Xu X, Gao L, Zhou J, Song Y, Hu J.	Zhou J	Front Immunol	2023	10.3389/fimmu.2023.1089026
24	Therapeutic drug monitoring guided dosing versus standard dosing of alectinib in advanced ALK positive non-small cell lung cancer patients: Study protocol for an international, multicenter phase IV randomized controlled trial (ADAPT ALEC)	Case reports, guidelines, protocol and meeting abstracts	Meertens M, Muntinghe-Wagenaar MB, Sikkema BJ, Lopez-Yurda M, Retèl VP, Paats MS, Ter Heine R, Schuuring E, Timens W, Touw DJ, van Boven JFM, de Langen AJ, Hashemi SMS, Hendriks LEL, Croes S, van den Heuvel MM, Dingemans AC, Mathijssen RHJ, Smit EF, Huitema ADR, Steeghs N, van der Wekken AJ.	Meertens M	Front Oncol	2023	10.3389/fonc.2023.1136221
25	Expert Consensus Recommendations on Biomarker Testing in Metastatic and Nonmetastatic NSCLC in Asia	Reviews	Mitsudomi T, Tan D, Yang JC, Ahn MJ, Batra U, Cho BC, Cornelio G, Lim T, Mok T, Prabhash K, Reungwetwattana T, Ren SX, Singh N, Toyooka S, Wu YL, Yang PC, Yatabe Y.	Mitsudomi T	J Thorac Oncol	2023	10.1016/j.jtho.2022.10.021
26	A case of granulocyte colony-stimulating factor producing lung adenocarcinoma with anaplastic lymphoma kinase gene rearrangements	Case reports, guidelines, protocol and meeting abstracts	Choi KJ, Kim KC, Kim EJ.	Choi KJ	Respir Med Case Rep	2023	10.1016/j.rmcr.2023.101885
27	Remarkable Clinical Response of ALK-Rearranged/TP53-Mutant Lung Adenocarcinoma with Liver Metastasis to Atezolizumab-Bevacizumab-Carboplatin-Paclitaxel After ALK Inhibitors: A Case Report	Case reports, guidelines, protocol and	Iso H, Miyanaga A, Kadoma N, Shinbu K, Tozuka T, Murata A, Nishima S, Sato Y, Nakamichi S, Matsumoto M, Noro R, Terasaki Y, Kubota K, Seike M.	Iso H	Onco Targets Ther	2023	10.2147/OTT.S404035

		meeting abstracts					
28	Entrectinib Response to ROS1-Fusion-Positive Non-Small-Cell Lung Cancer That Progressed on Crizotinib with Leptomeningeal Metastasis: A Case Report	Case reports, guidelines, protocol and meeting abstracts	Sawada H, Taniguchi Y, Iizuka S, Ikeda T, Aga M, Hamakawa Y, Miyazaki K, Misumi Y, Agemi Y, Nakamura Y, Maeda K, Shimokawa T, Okamoto H.	Sawada H	Case Rep Oncol	2023	10.1159/000534549
29	The safety and efficacy for the treatment of alectinib in a women with ALK-positive lung cancer delivered a healthy male neonate throughout Pregnancy: A case report	Case reports, guidelines, protocol and meeting abstracts	Shang M, Luo X, Wu J, Wang Z, Chen Q, Zhou Y.	Shang M	Lung Cancer	2023	10.1016/j.lungcan.2023.107188
30	Targeted treatments after chemoradiotherapy failure in a patient with relapsed, advanced non- small cell lung cancer with on- therapy circulating tumor biomarker monitoring: A case report	Case reports, guidelines, protocol and meeting abstracts	Bi Y, Xia C, Zhang X, Liu H.	Bi Y	Oncol Lett	2023	10.3892/ol.2023.13993
31	Retrospective Assessment of Complementary Liquid Biopsy on Tissue Single-Gene Testing for Tumor Genotyping in Advanced NSCLC	Reviews	Desmeules P, Dusselier M, Bouffard C, Bafaro J, Fortin M, Labbé C, Joubert P.	Desmeules P	Curr Oncol	2023	10.3390/curroncol30010045
32	Development of HPLC-MS/MS assay for quantitation of ensartinib in human plasma and its application to a pharmacokinetics study in Chinese patients	Reviews	Li H, Wang Y, Chen X, Chen C, Cui J, Han Y, Ding L.	Li H	Biomed Chromatogr	2023	10.1002/bmc.5610
33	Transformation of ALK-positive NSCLC to SCLC after alectinib resistance and response to combined atezolizumab: a case report	Case reports, guidelines, protocol and meeting abstracts	Xia G, Huang J, Ni J, Song M, Zhang J, Hofman P, Christopoulos P, Grenda A, Huang M.	Xia G	Transl Lung Cancer Res	2023	10.21037/tlcr-23-154
34	Diverse Resistant Mechanisms Identified Using Serial Next-Generation Sequencing in a Patient With ALK-Rearranged Metastatic Lung Adenocarcinoma: A Case Report	Case reports, guidelines, protocol and meeting abstracts	Saw SPL, Lim DW.	Saw SPL	JTO Clin Res Rep	2023	10.1016/j.jtocrr.2023.100512
35	EML4-ALK rearrangement of lung large cell neuroendocrine carcinoma: a case report	Data not related or missing	Chen D, Ma S, Sun L, Lang Y, Yang B.	Chen D	Ann Transl Med	2023	10.21037/atm-22-6062
36	Prediction of Drug-Drug Interactions with Ensartinib as a Time-Dependent CYP3A Inhibitor Using Physiologically Based Pharmacokinetic Model	Data not related or missing	Wang X, Yu Y, Liu H, Bu F, Shen C, He Q, Zhu X, Jiang P, Han B, Xiang X.	Wang X	Drug Metab Dispos	2023	10.1124/dmd.123.001373
37	Impact of Tyrosine Kinase Inhibitors on the Immune Response to SARS-CoV-2 Vaccination in Patients with Non-Small Cell Lung Cancer	Data not related or missing	Hernández-Pedro N, Arroyo-Hernández M, Barrios-Bernal P, Romero-Nuñez E, Sosa-Hernandez VA, Ávila-Ríos S, Maravillas-Montero JL, Pérez-Padilla R, de Miguel-Perez D, Rolfo C, Arrieta O.	Hernández-Pedro N	Vaccines (Basel)	2023	10.3390/vaccines11101612
38	Molecular patterns of egyptian patients with non-squamous non-small-cell lung cancers: a clinicopathological study	Data not related or missing	Ismail MS, Kassem L, Ali AA, Ahmed FE, Shalaby M, Magdy S.	Ismail MS	J Egypt Natl Canc Inst	2023	10.1186/s43046-023-00167-2
39	A Phase 1 Drug-Drug Interaction Study Between Brigatinib and the CYP3A Substrate Midazolam in Patients With ALK-Positive or ROS1-Positive Solid Tumors	Data not related or missing	Hanley MJ, D'Arcangelo M, Felip E, Garrido P, Zhu J, Ye M, Vranceanu F, Gupta N.	Hanley MJ	J Clin Pharmacol	2023	10.1002/jcph.2198
40	Liquid biopsy detects genomic drivers in NSCLC without EGFR mutations by single-plex testing: WJOG13620L	Data not related or missing	Uemura T, Kenmotsu H, Hazama D, Teraoka S, Kobe H, Azuma K, Yamaguchi T, Masuda T, Yokoyama T, Otsubo K, Haratani K, Hayakawa D, Oki M, Takemoto S, Ozaki T, Akashi Y, Hata A, Hashimoto H, Yamamoto N, Nakagawa K.	Uemura T	Cancer Med	2023	10.1002/cam4.6668

41	Preclinical evidence for anaplastic lymphoma kinase inhibitors as novel therapeutic treatments for cholangiocarcinoma	Data not related or missing	Myint KZ, Sueca-Comes M, Collier P, Balasubramanian B, Venkatraman S, Gordan J, Zaitoun AM, Mukherjee A, Arora A, Larbcharoensub N, Suriyonplengsaeng C, Wongprasert K, Janvilisri T, Gomez D, Grabowska AM, Tohtong R, Bates DO, Yacqub-Usman K.	Myint KZ	Front Oncol	2023	10.3389/fonc.2023.1184900
42	Molecular characteristics and prognostic factors of leptomeningeal metastasis in non-small cell lung cancer	Data not related or missing	Liu X, Li G, Zhang H, Chang Q, Fang M, Lu C, Tian P, Mei F.	Liu X	Clin Neurol Neurosurg	2023	10.1016/j.clineuro.2022.107572
43	Tyrosine Kinase Inhibitors Target B Lymphocytes	Data not related or missing	Upfold NLE, Petakh P, Kamyshnyi A, Oksenych V.	Upfold NLE	Biomolecules	2023	10.3390/biom13030438
44	Prediction of trough concentration and ALK occupancy in plasma and cerebrospinal fluid using physiologically based pharmacokinetic modeling of crizotinib, alectinib, and lorlatinib	Data not related or missing	Li B, Liu S, Feng H, Du C, Wei L, Zhang J, Jia G, Wu C.	Li B	Front Pharmacol	2023	10.3389/fphar.2023.1234262
45	Plasma versican and plasma exosomal versican as potential diagnostic markers for non-small cell lung cancer	Data not related or missing	Chang W, Zhu J, Yang D, Shang A, Sun Z, Quan W, Li D.	Chang W	Respir Res	2023	10.1186/s12931-023-02423-4
46	Detecting ALK, ROS1, and RET fusions and the METΔex14 splicing variant in liquid biopsies of non-small-cell lung cancer patients using RNA-based techniques	Data not related or missing	Giménez-Capitán A, Sánchez-Herrero E, Robado de Lope L, Aguilar-Hernández A, Sullivan I, Calvo V, Moya-Horno I, Viteri S, Cabrera C, Aguado C, Armiger N, Valarezo J, Mayo-de-Las-Casas C, Reguart N, Rosell R, Provencio M, Romero A, Molina-Vila MA.	Giménez-Capitán A	Mol Oncol	2023	10.1002/1878-0261.13468
47	Detection of resistance mutations in patients with anaplastic lymphoma kinase-rearranged lung cancer through liquid biopsy	Data not related or missing	Sasaki T, Yoshida R, Nitanai K, Watanabe T, Tenma T, Kida R, Mori C, Umekage Y, Hirai N, Minami Y, Okumura S.	Sasaki T	Transl Lung Cancer Res	2023	10.21037/tlcr-22-671
48	Inflammatory myofibroblastic tumor of the lung involving the central nervous system in pediatric patients	Data not related or missing	Ramos JG, Ochoa A, Cicutti S, Del Rio R, Lubienieki F.	Ramos JG	Childs Nerv Syst	2023	10.1007/s00381-023-05925-0
49	Targeted Therapy and Immunotherapy in Early-Stage Non-Small Cell Lung Cancer: Current Evidence and Ongoing Trials	Reviews	de Scordilli M, Michelotti A, Bertoli E, De Carlo E, Del Conte A, Bearz A.	de Scordilli M	Int J Mol Sci	2022	10.3390/ijms23137222
50	Precision medicine in non-small cell lung cancer: Current applications and future directions	Reviews	Yang SR, Schultheis AM, Yu H, Mandelker D, Ladanyi M, Büttner R.	Yang SR	Semin Cancer Biol	2022	10.1016/j.semcancer.2020.07.009
51	Efficacy of Brigatinib in Patients With Advanced ALK-Positive NSCLC Who Progressed on Alectinib or Ceritinib: ALK in Lung Cancer Trial of brigAtinib-2 (ALTA-2)	Data not related or missing	Ou SI, Nishio M, Ahn MJ, Mok T, Barlesi F, Zhou C, Felip E, de Marinis F, Kim SW, Pérol M, Liu G, Migliorino MR, Kim DW, Novello S, Bearz A, Garrido P, Mazieres J, Morabito A, Lin HM, Yang H, Niu H, Zhang P, Kim ES.	Ou SI	J Thorac Oncol	2022	10.1016/j.jtho.2022.08.018
52	Anaplastic lymphoma kinase inhibitors and their effect on the kidney	Reviews	Bonilla M, Jhaveri KD, Izzedine H.	Bonilla M	Clin Kidney J	2022	10.1093/ckj/sfac062
53	Applications of Liquid Biopsies in Non-Small-Cell Lung Cancer	Reviews	Pesta M, Shetti D, Kulda V, Knizkova T, Houfkova K, Bagheri MS, Svaton M, Polivka J.	Pesta M	Diagnostics (Basel)	2022	10.3390/diagnostics12081799
54	Prognostic Value of Circulating Tumor DNA (ctDNA) in Oncogene-Driven NSCLC: Current Knowledge and Future Perspectives	Reviews	Zografos E, Dimitrakopoulos FI, Koutras A.	Zografos E	Cancers (Basel)	2022	10.3390/cancers14194954
55	Longitudinal monitoring of cell-free DNA methylation in ALK-positive non-small cell lung cancer patients	Data not related or missing	Janke F, Angeles AK, Riediger AL, Bauer S, Reck M, Stenzinger A, Schneider MA, Muley T, Thomas M, Christopoulos P, Sültmann H.	Janke F	Clin Epigenetics	2022	10.1186/s13148-022-01387-4
56	Liquid Biopsy Analysis as a Tool for TKI-Based Treatment in Non-Small Cell Lung Cancer	Reviews	Buszka K, Ntzifa A, Owecka B, Kamińska P, Kolecka-Bednarczyk A, Zabel M, Nowicki M, Lianidou E, Budna-Tukan J.	Buszka K	Cells	2022	10.3390/cells11182871
57	Fibroblast growth factor receptor 3 overexpression mediates ALK inhibitor resistance in ALK-rearranged non-small cell lung cancer	Data not related or missing	Sakashita T, Yanagitani N, Koike S, Low SK, Takagi S, Baba S, Takeuchi K, Nishio M, Fujita N, Katayama R.	Sakashita T	Cancer Sci	2022	10.1111/cas.15529
58	Circulating Cell-free DNA as a Prognostic Biomarker in Patients with Advanced ALK+ Non-small Cell Lung Cancer in the Global Phase III ALEX Trial	Data not related or missing	Dziadziuszko R, Peters S, Mok T, Camidge DR, Gadgeel SM, Ou SI, Konopa K, Noé J, Nowicka M, Bordogna W, Morcos PN, Smoljanovic V, Shaw AT.	Dziadziuszko	Clin Cancer Res	2022	10.1158/1078-0432.CCR-21-2840

				R			
59	Cerebrospinal fluid-derived circulating tumor DNA is more comprehensive than plasma in NSCLC patients with leptomeningeal metastases regardless of extracranial evolution	Reviews	Yang H, Wen L, Zhao C, Chen J, Zhou Z, Zhou C, Cai L, Zhou C.	Yang H	Heliyon	2022	10.1016/j.heliyon.2022.e12374
60	Concordance of Genomic Profiles in Matched Tissue and Plasma Samples From Chinese Patients With Lung Cancer	Reviews	He Y, Guo W, Xu M, Huang J, Zhang X, Su H, Hong D, Liu Q.	He Y	Clin Med Insights Oncol	2022	10.1177/11795549221116834
61	EGFR detection by liquid biopsy: ripe for clinical usage	Reviews	Batra U, Nathany S, Sharma M, Jain P, Dhanda S, Singh H, Jain A, Mehta A.	Batra U	Future Oncol	2022	10.2217/fon-2021-0620
62	Real-world circulating tumor DNA analysis depicts resistance mechanism and clonal evolution in ALK inhibitor-treated lung adenocarcinoma patients	Data not related or missing	Hua G, Zhang X, Zhang M, Wang Q, Chen X, Yu R, Bao H, Liu J, Wu X, Shao Y, Liang B, Lu K.	Hua G	ESMO Open	2022	10.1016/j.esmoop.2021.100337
63	Successful treatment of refractory brain metastases from ALK-positive lung cancer with lorlatinib	Case reports, guidelines, protocol and meeting abstracts	Nakagawa Y, Shimizu T, Hiranuma H, Gon Y.	Nakagawa Y	Thorac Cancer	2022	10.1111/1759-7714.14406
64	Evaluation of Lorlatinib Cerebrospinal Fluid Concentrations in Relation to Target Concentrations for Anaplastic Lymphoma Kinase (ALK) Inhibition	Reviews	Sun S, Pithavala YK, Martini JF, Chen J.	Sun S	J Clin Pharmacol	2022	10.1002/jcph.2056
65	Conteltinib (CT-707) in patients with advanced ALK-positive non-small cell lung cancer: a multicenter, open-label, first-in-human phase 1 study	Data not related or missing	Xing P, Zhao Q, Zhang L, Wang H, Huang D, Hu P, Sun Y, Shi Y.	Xing P	BMC Med	2022	10.1186/s12916-022-02646-0
66	Phase II Trial of the Combination of Alectinib with Bevacizumab in Alectinib Refractory ALK-Positive Nonsquamous Non-Small-Cell Lung Cancer (NLCTG1501)	Data not related or missing	Watanabe S, Sakai K, Matsumoto N, Koshio J, Ishida A, Abe T, Ishikawa D, Tanaka T, Aoki A, Kajiwara T, Koyama K, Miura S, Goto Y, Sekiya T, Suzuki R, Kushiro K, Fujisaki T, Yanagimura N, Ohtsubo A, Shoji S, Nozaki K, Saida Y, Yoshizawa H, Nishio K, Kikuchi T.	Watanabe S	Cancers (Basel)	2022	10.3390/cancers15010204
67	The quantum leap in therapeutics for advanced ALK+ non-small cell lung cancer and pursuit to cure with precision medicine	Reviews	Itchins M, Pavlakis N.	Itchins M	Front Oncol	2022	10.3389/fonc.2022.959637
68	Longitudinal monitoring by next-generation sequencing of plasma cell-free DNA in ALK rearranged NSCLC patients treated with ALK tyrosine kinase inhibitors	Data not related or missing	Kwon M, Ku BM, Olsen S, Park S, Lefterova M, Odegaard J, Jung HA, Sun JM, Lee SH, Ahn JS, Park K, Ahn MJ.	Kwon M	Cancer Med	2022	10.1002/cam4.4663
69	Efficacy and safety of alectinib in ALK-positive non-small cell lung cancer and blood markers for prognosis and efficacy: a retrospective cohort study	Data not related or missing	Jiang Y, Shi Y, Liu Y, Wang Z, Ma Y, Shi X, Lu L, Wang Z, Li H, Zhang Y, Liu C, Zhang S, Zhong Z, Lu J, Shi M, Shen B, Zhou G, Yin R, Galetta D, Grenda A, Romero A, Hughes BGM, Chen C, Wang X, Feng J.	Jiang Y	Transl Lung Cancer Res	2022	10.21037/tlcr-22-857
70	AGE/RAGE axis regulates reversible transition to quiescent states of ALK-rearranged NSCLC and pancreatic cancer cells in monolayer cultures	Data not related or missing	Kadonosono T, Miyamoto K, Sakai S, Matsuo Y, Kitajima S, Wang Q, Endo M, Niibori M, Kuchimaru T, Soga T, Hirota K, Kizaka-Kondoh S.	Kadonosono T	Sci Rep	2022	10.1038/s41598-022-14272-0
71	Final efficacy and safety data, and exploratory molecular profiling from the phase III ALUR study of alectinib versus chemotherapy in crizotinib-pretreated ALK-positive non-small-cell lung cancer	Data not related or missing	Wolf J, Helland Å, Oh IJ, Migliorino MR, Dziadziuszko R, Wrona A, de Castro J, Mazieres J, Griesinger F, Chlistalla M, Cardona A, Ruf T, Trunzer K, Smoljanovic V, Novello S.	Wolf J	ESMO Open	2022	10.1016/j.esmoop.2021.100333
72	Audit of Molecular Mechanisms of Primary and Secondary Resistance to Various Generations of Tyrosine Kinase Inhibitors in Known Epidermal Growth Factor Receptor-Mutant Non-small Cell Lung Cancer Patients in a Tertiary Centre	Reviews	Suryavanshi M, Jaipuria J, Mattoo S, Dhandha S, Khatri M.	Suryavanshi M	Clin Oncol (R Coll Radiol)	2022	10.1016/j.clon.2022.06.003
73	Silibinin Suppresses the Hyperlipidemic Effects of the ALK-Tyrosine Kinase Inhibitor Lorlatinib in Hepatic Cells	Data not related or missing	Verdura S, Encinar JA, Fernández-Arroyo S, Joven J, Cuyàs E, Bosch-Barrera J, Menendez JA.	Verdura S	Int J Mol Sci	2022	10.3390/ijms23179986
74	Case Report: Pathological Complete Response to Neoadjuvant Alectinib in a Patient With Resectable ALK-Positive Non-Small Cell Lung Cancer	Case reports, guidelines, protocol and meeting abstracts	Hu Y, Ren S, Wang R, Han W, Xiao P, Wang L, Yu F, Liu W.	Hu Y	Front Pharmacol	2022	10.3389/fphar.2022.816683
7	The Role of Serial Liquid Biopsy in the Management of Metastatic Non-Small Cell Lung	Case reports,	Sama S, Le T, Ullah A, Elhelf IA, Kavuri SK, Karim NA.	Sama S	Clin Pract	2022	10.3390/clinpract120300

5	Cancer (NSCLC)	guidelines, protocol and meeting abstracts					46
76	Predicting EGFR mutation, ALK rearrangement, and uncommon EGFR mutation in NSCLC patients by driverless artificial intelligence: a cohort study	Data not related or missing	Tan X, Li Y, Wang S, Xia H, Meng R, Xu J, Duan Y, Li Y, Yang G, Ma Y, Jin Y.	Tan X	Respir Res	2022	10.1186/s12931-022-02053-2
77	Effectiveness of alectinib and osimertinib in a brain metastasized lung adenocarcinoma patient with concurrent EGFR mutations and DCTN1-ALK fusion	Data not related or missing	Yin Q, Guo T, Zhou Y, Sun L, Meng M, Ma L, Wang X.	Yin Q	Thorac Cancer	2022	10.1111/1759-7714.14291
78	Classical ALK G1202R resistance mutation was identified in a lung adenocarcinoma patient with rare LOC388942-ALK fusion after sequential treatment with ALK-TKIs and anlotinib: a case report	Case reports, guidelines, protocol and meeting abstracts	Zhai X, Liu Y, Liang Z, Wang W, Qin T, Liu SV, Um SW, Luo F, Liu J.	Zhai X	Ann Transl Med	2022	10.21037/atm-22-5194
79	Role of chemokine-mediated angiogenesis in resistance towards crizotinib and its reversal by anlotinib in EML4-ALK positive NSCLC	Data not related or missing	Wang S, Lou N, Luo R, Hao X, Liu Y, Wang L, Shi Y, Han X.	Wang S	J Transl Med	2022	10.1186/s12967-022-03451-2
80	Pharmacokinetics of alectinib and its metabolite M4 in a patient with advanced lung adenocarcinoma undergoing hemodialysis: A case report	Case reports, guidelines, protocol and meeting abstracts	Park JE, Yoon YR, Kim CH, Lee J.	Park JE	Thorac Cancer	2022	10.1111/1759-7714.14357
81	Immune checkpoint inhibitor-related pneumonitis with atypical radiologic features in a patient with anti-aminoacyl-tRNA synthetase antibody	Case reports, guidelines, protocol and meeting abstracts	Ichihara S, Ogino H, Yoneda H, Haji K, Kagawa K, Murakami K, Mima M, Aoi Y, Mitsuhashi A, Tsukazaki Y, Yabuki Y, Ozaki R, Sato S, Nokihara H, Nishioka Y.	Ichihara S	Respir Med Case Rep	2022	10.1016/j.rmcr.2022.101797
82	Liquid Biopsy Testing for the Management of Patient with Non-Small Cell Lung Cancer Carrying a Rare Exon-20 EGFR Insertion	Data not related or missing	Morabito A, Manzo A, Montanino A, Rachiglio AM, Sforza V, Pasquale R, Costanzo R, Maiello MR, Sandomenico C, Gallo M, Palumbo G, De Luca A, La Rocca A, Martucci N, De Cecio R, Picone C, Lastoria S, Normanno N.	Morabito A	Oncologist	2022	10.1093/oncolo/oyab002
83	The diagnostic importance of pathogenic variants and variant coexistence determined by NGS-based liquid biopsy approach in patients with lung adenocarcinoma	Data not related or missing	Cine N, Ali Sen E, Demir G, Gokbayrak M, Guzdolu E, Sertdemir N, Aydin D, Kurtas O, Reka S, Sunnetci-Akkoyunlu D, Eren-Keskin S, Uygun K, Cabuk D, Gorkem Aksu M, Zafer Canturk N, Savli H.	Cine N	Mol Cell Probes	2022	10.1016/j.mcp.2022.101819
84	Acquired G2032R Resistance Mutation in ROS1 to Lorlatinib Therapy Detected with Liquid Biopsy	Data not related or missing	Jóri B, Falk M, Hövel I, Weist P, Tiemann M, Heukamp LC, Griesinger F.	Jóri B	Curr Oncol	2022	10.3390/curroncol29090520
85	A Phase I Study to Evaluate the Pharmacokinetics and Safety of Lorlatinib in Adults with Mild, Moderate, and Severe Renal Impairment	Data not related or missing	Lin S, Gong J, Canas GC, Winkle P, Pelletier K, LaBadie RR, Ginman K, Pithavala YK.	Lin S	Eur J Drug Metab Pharmacokinet	2022	10.1007/s13318-021-00747-4
86	WX-0593 combined with an epithelial growth factor receptor (EGFR) monoclonal antibody in the treatment of xenograft tumors carrying triple EGFR mutations	Data not related or missing	Zheng Q, Chen D, Wang X, Yang Y, Zhao S, Dong X, Ma C, Zhang X, Duan H, Sun Y, Zheng S.	Zheng Q	Ann Transl Med	2022	10.21037/atm-22-2780
87	Expression of SARS-CoV-2-Related Surface Proteins in Non-Small-Cell Lung Cancer Patients and the Influence of Standard of Care Therapy	Data not related or missing	Deben C, Le Compte M, Siozopoulou V, Lambrechts H, Hermans C, Lau HW, Huizing M, Lamote K, Hendriks JMH, Van Dam P, Pauwels P, Smits ELJ, Peeters M, Lardon F.	Deben C	Cancers (Basel)	2022	10.3390/cancers14174074
88	Increased blood-based intratumor heterogeneity (bITH) is associated with unfavorable outcomes of immune checkpoint inhibitors plus chemotherapy in non-small cell lung cancer	Data not related or missing	Zhou J, Bao M, Gao G, Cai Y, Wu L, Lei L, Zhao J, Ji X, Huang Y, Su C.	Zhou J	BMC Med	2022	10.1186/s12916-022-02444-8
89	Comprehensive characterization reveals sputum supernatant as a valuable alternative liquid biopsy for genome profiling in advanced non-small cell lung cancer	Data not related or missing	Xie X, Wu J, Guo B, Wang L, Deng H, Lin X, Liu M, Qin Y, Luo W, Yang Y, Zou X, Hou T, Xiang J, Chen Z, Zhou C.	Xie X	Respir Res	2022	10.1186/s12931-022-02097-4

90	ALK-Fusion Transcripts Can Be Detected in Extracellular Vesicles (EVs) from Nonsmall Cell Lung Cancer Cell Lines and Patient Plasma: Toward EV-Based Noninvasive Testing	Data not related or missing	Sánchez-Herrero E, Campos-Silva C, Cáceres-Martell Y, Robado de Lope L, Sanz-Moreno S, Serna-Blasco R, Rodríguez-Festa A, Ares Trotta D, Martín-Acosta P, Patiño C, Coronado MJ, Beneitez A, Jara R, Lago-Baameiro N, Camino T, Cruz-Bermúdez A, Pardo M, González-Rumayor V, Valés-Gómez M, Provencio M, Romero A.	Sánchez-Herrero E	Clin Chem	2022	10.1093/clinchem/hvac021
91	Monitoring Circulating Tumor DNA in Untreated Non-Small-Cell Lung Cancer Patients	Data not related or missing	Ryu WK, Oh S, Lim JH, Lee SJ, Shin HT, Ryu JS.	Ryu WK	Int J Mol Sci	2022	10.3390/ijms23179527
92	The Pan-Tumor Landscape of Targetable Kinase Fusions in Circulating Tumor DNA	Data not related or missing	Lee JK, Hazar-Rethinam M, Decker B, Gjoerup O, Madison RW, Lieber DS, Chung JH, Schrock AB, Creeden J, Venstrom J, Alexander B, Oxnard GR.	Lee JK	Clin Cancer Res	2022	10.1158/1078-0432.CCR-21-2136
93	Therapeutic Sequencing in ALK(+) NSCLC	Case reports, guidelines, protocol and meeting abstracts	Elsayed M, Christopoulos P.	Elsayed M	Pharmaceuticals (Basel)	2021	10.3390/ph14020080
94	Predictive and Prognostic Biomarkers for Lung Cancer Bone Metastasis and Their Therapeutic Value	Reviews	Chai X, Yinwang E, Wang Z, Wang Z, Xue Y, Li B, Zhou H, Zhang W, Wang S, Zhang Y, Li H, Mou H, Sun L, Qu H, Wang F, Zhang Z, Chen T, Ye Z.	Chai X	Front Oncol	2021	10.3389/fonc.2021.692788
95	New Advances in Liquid Biopsy Technologies for Anaplastic Lymphoma Kinase (ALK)-Positive Cancer	Reviews	Villa M, Sharma GG, Manfroni C, Cortinovis D, Mologni L.	Villa M	Cancers (Basel)	2021	10.3390/cancers13205149
96	Systemic Therapy for Lung Cancer Brain Metastases	Reviews	Pellerino A, Bruno F, Rudà R, Soffietti R.	Pellerino A	Curr Treat Options Oncol	2021	10.1007/s11864-021-00911-7
97	Liquid biopsy for ALK-positive early non-small-cell lung cancer predicts disease relapse	Data not related or missing	Li J, Dong W, Liu LN, Huang YJ, Xiao MF.	Li J	Future Oncol	2021	10.2217/fon-2020-0554
98	Earlier extracranial progression and shorter survival in ALK-rearranged lung cancer with positive liquid rebiopsies	Data not related or missing	Christopoulos P, Dietz S, Angeles AK, Rheinheimer S, Kazdal D, Volckmar AL, Janke F, Endris V, Meister M, Kriegsmann M, Zemojtel T, Reck M, Stenzinger A, Thomas M, Sültmann H.	Christopoulos P	Transl Lung Cancer Res	2021	10.21037/tlcr-21-32
99	Early identification of disease progression in ALK-rearranged lung cancer using circulating tumor DNA analysis	Reviews	Angeles AK, Christopoulos P, Yuan Z, Bauer S, Janke F, Ogrodnik SJ, Reck M, Schlesner M, Meister M, Schneider MA, Dietz S, Stenzinger A, Thomas M, Sültmann H.	Angeles AK	NPJ Precis Oncol	2021	10.1038/s41698-021-00239-3
100	SPACEWALK: A Remote Participation Study of ALK Resistance Leveraging Plasma Cell-Free DNA Genotyping	Data not related or missing	Lawrence MN, Tamen RM, Martinez P, Sable-Hunt A, Addario T, Barbour P, Shaffer T, Hosseini SA, Bertucci C, Lim LP, Hong F, Michael K, Simon GR, Riess JW, Awad MM, Oxnard GR.	Lawrence MN	JTO Clin Res Rep	2021	10.1016/j.jtocrr.2021.100151
101	Decoding the Evolutionary Response to Ensartinib in Patients With ALK-Positive NSCLC by Dynamic Circulating Tumor DNA Sequencing	Data not related or missing	Yang Y, Huang J, Wang T, Zhou J, Zheng J, Feng J, Zhuang W, Chen J, Zhao J, Zhong W, Zhao Y, Zhang Y, Song Y, Hu Y, Yu Z, Gong Y, Chen Y, Ye F, Zhang S, Cao L, Fan Y, Wu G, Guo Y, Zhou C, Ma K, Fang J, Feng W, Liu Y, Zheng Z, Li G, Wang H, Cang S, Wu N, Song W, Liu X, Zhao S, Ding L, Mao L, Selvaggi G, Zhu L, Xiao S, Yuan X, Shen Z, Zhang L.	Yang Y	J Thorac Oncol	2021	10.1016/j.jtho.2021.01.1615
102	TPX-0131, a Potent CNS-penetrant, Next-generation Inhibitor of Wild-type ALK and ALK-resistant Mutations	Data not related or missing	Murray BW, Zhai D, Deng W, Zhang X, Ung J, Nguyen V, Zhang H, Barrera M, Parra A, Cowell J, Lee DJ, Aloysius H, Rogers E.	Murray BW	Mol Cancer Ther	2021	10.1158/1535-7163.MCT-21-0221
103	Clinical utility of next-generation sequencing-based ctDNA testing for common and novel ALK fusions	Reviews	Mondaca S, Lebow ES, Namakydoust A, Razavi P, Reis-Filho JS, Shen R, Offin M, Tu HY, Murciano-Goroff Y, Xu C, Makhnin A, Martinez A, Pavlakis N, Clarke S, Itchins M, Lee A, Rimner A, Gomez D, Rocco G, Chaft JE, Riely GJ, Rudin CM, Jones DR, Li M, Shaffer T, Hosseini SA, Bertucci C, Lim LP, Drilon A, Berger MF, Benayed R, Arcila ME, Isbell JM, Li BT.	Mondaca S	Lung Cancer	2021	10.1016/j.lungcan.2021.06.018
104	Alectinib for Miliary Lung Metastasis in ALK-Positive Lung Adenocarcinoma	Case reports, guidelines, protocol and meeting	Satoh H, Okuma Y, Kashima J, Konnno-Yamamoto A, Yatabe Y, Ohe Y.	Satoh H	Onco Targets Ther	2021	10.2147/OTT.S300229

		abstracts					
105	ALK Rearrangement in Small-Cell Lung Cancer and Durable Response to Alectinib: A Case Report	Case reports, guidelines, protocol and meeting abstracts	Sun N, Zhuang Y, Zhang J, Chen S, Dai Y, Guo R.	Sun N	Onco Targets Ther	2021	10.2147/OTT.S323700
106	Detection of ALK fusion transcripts in plasma of non-small cell lung cancer patients using a novel RT-PCR based assay	Data not related or missing	Heeke S, Benzaquen J, Vallee A, Allegra M, Mazieres J, Fayada J, Rajamani J, Lee M, Ordinario E, Tiotiu A, Cadranel J, Poudenx M, Moro-Sibilot D, Barlesi F, Gervais R, Thariat J, Tanga V, Boutros J, Ilié M, Hofman V, Marquette CH, Denis MG, Hofman P.	Heeke S	Ann Transl Med	2021	10.21037/atm-20-7900
107	Unique Genomic Alterations of Cerebrospinal Fluid Cell-Free DNA Are Critical for Targeted Therapy of Non-Small Cell Lung Cancer With Leptomeningeal Metastasis	Reviews	Wang Y, Jiang F, Xia R, Li M, Yao C, Li Y, Li H, Zhao Q, Shi M, Yu Y, Shao YW, Zhou G, Xia H, Miao L, Cai H.	Wang Y	Front Oncol	2021	10.3389/fonc.2021.701171
108	Clinical value of serum albumin level in patients with non-small cell lung cancer and anaplastic lymphoma kinase (ALK) rearrangement	Reviews	Zhang X, Xing P, Hao X, Li J.	Zhang X	Ann Palliat Med	2021	10.21037/apm-21-3379
109	One-Step Polymerase Chain Reaction-Free Nanowire-Based Plasma Cell-Free DNA Assay to Detect EML4-ALK Fusion and to Monitor Resistance in Lung Cancer	Data not related or missing	Lee Y, Cho Y, Park EY, Park SY, Hwang KH, Han JY.	Lee Y	Oncologist	2021	10.1002/onco.13902
110	Brigatinib versus other second-generation ALK inhibitors as initial treatment of anaplastic lymphoma kinase positive non-small cell lung cancer with deep phenotyping: study protocol of the ABP trial	Case reports, guidelines, protocol and meeting abstracts	Christopoulos P, Bozorgmehr F, Brückner L, Chung I, Krisam J, Schneider MA, Stenzinger A, Eickhoff R, Mueller DW, Thomas M.	Christopoulos P	BMC Cancer	2021	10.1186/s12885-021-08460-w
111	Pharmacoenhancement of Low Crizotinib Plasma Concentrations in Patients with Anaplastic Lymphoma Kinase-Positive Non-Small Cell Lung Cancer using the CYP3A Inhibitor Cobicistat	Case reports, guidelines, protocol and meeting abstracts	Hohmann N, Bozorgmehr F, Christopoulos P, Mikus G, Blank A, Burhenne J, Thomas M, Haefeli WE.	Hohmann N	Clin Transl Sci	2021	10.1111/cts.12921
112	Pharmacokinetics of Lorlatinib After Single and Multiple Dosing in Patients with Anaplastic Lymphoma Kinase (ALK)-Positive Non-Small Cell Lung Cancer: Results from a Global Phase I/II Study	Data not related or missing	Chen J, O'Gorman MT, James LP, Klamerus KJ, Mugundu G, Pithavala YK.	Chen J	Clin Pharmacokinet	2021	10.1007/s40262-021-01015-z
113	Clinical efficacy and safety of crizotinib and alectinib in ALK-positive non-small cell lung cancer treatment and predictive value of CEA and CA125 for treatment efficacy	Data not related or missing	Li Z, Zhao J.	Li Z	Am J Transl Res	2021	https://pmc.ncbi.nlm.nih.gov/articles/PMC8661162/
114	Next Generation Sequencing in the Management of Leptomeningeal Metastases of Non-Small Cell Lung Cancer: A Case Report and Literature Review	Reviews	Li S, Ke L, Meng X, Zhou H, Zhang X, Wu H, Yu J, Zhang H.	Li S	Recent Pat Anticancer Drug Discov	2021	10.2174/1574892815666201127114224
115	Colorectal Cancer with EML4-ALK Fusion Gene Response to Alectinib: A Case Report and Review of the Literature	Case reports, guidelines, protocol and meeting abstracts	Hsiao SY, He HL, Weng TS, Lin CY, Chao CM, Huang WT, Tsao CJ.	Hsiao SY	Case Rep Oncol	2021	10.1159/000511069
116	Exceptional response to the ALK and ROS1 inhibitor lorlatinib and subsequent mechanism of resistance in relapsed ALK F1174L-mutated neuroblastoma	Data not related or missing	Liu T, Merguerian MD, Rowe SP, Pratilas CA, Chen AR, Ladle BH.	Liu T	Cold Spring Harb Mol	2021	10.1101/mcs.a006064

					Case Stud		
117	Acquired ALK Resistance Mutations Identified from Liquid Biopsy in an ALK-Rearranged Squamous Cell Lung Cancer Patient Treated with Sequential ALK TKI Therapy: A Case Report	Case reports, guidelines, protocol and meeting abstracts	Yao B, Han X, Pang L, Xu C, Liu S, Cheng X, Chen J.	Yao B	Onco Targets Ther	2021	10.2147/OTT.S315832
118	A novel GHR-ALK fusion gene in a patient with metastatic lung adenocarcinoma and its response to crizotinib: a case report	Case reports, guidelines, protocol and meeting abstracts	Pan X, Zhong A, Xing Y, Li X, Du H, Shi M.	Pan X	J Int Med Res	2021	10.1177/03000605211044652
119	Successful treatment with alectinib after crizotinib-induced hepatitis in ALK-rearranged advanced lung cancer patient: a case report	Case reports, guidelines, protocol and meeting abstracts	Duarte FA, Rodrigues LB, Paes FR, Diniz PHC, Lima HFCA.	Duarte FA	BMC Pulm Med	2021	10.1186/s12890-020-01390-6
120	Coexistence of a secondary STRN-ALK, EML4-ALK double-fusion variant in a lung adenocarcinoma patient with EGFR mutation: a case report	Case reports, guidelines, protocol and meeting abstracts	Zeng Q, Gao H, Zhang L, Qin S, Gu Y, Chen Q.	Zeng Q	Anticancer Drugs	2021	10.1097/CAD.0000000000001094
121	Treatment of advanced lung cancer based on genomic profiling using liquid biopsy (plasma): A review of three cases	Case reports, guidelines, protocol and meeting abstracts	Mitsumura T, Kumaki Y, Takahashi K, Matsudera S, Sakakibara R, Honda T, Ishizuka M, Iijima Y, Shirai T, Okamoto T, Tateishi T, Sakashita H, Miyake S, Ikeda S, Miyazaki Y.	Mitsumura T	Thorac Cancer	2021	10.1111/1759-7714.14098
122	NGS-based liquid biopsy profiling identifies mechanisms of resistance to ALK inhibitors: a step toward personalized NSCLC treatment	Data not related or missing	Sánchez-Herrero E, Serna-Blasco R, Ivanchuk V, García-Campelo R, Dómine Gómez M, Sánchez JM, Massutí B, Reguart N, Camps C, Sanz-Moreno S, Calabuig-Fariñas S, Jantus-Lewintre E, Arnal M, Fernández-Orth D, Calvo V, González-Rumayor V, Provencio M, Romero A.	Sánchez-Herrero E	Mol Oncol	2021	10.1002/1878-0261.13033
123	Corrigendum to "Clinical utility of next-generation sequencing-based ctDNA testing for common and novel ALK fusions" [Lung Cancer 159 (2021) 66-73]	Data not related or missing	Mondaca S, Lebow ES, Namakydoust A, Razavi P, Reis-Filho JS, Shen R, Offin M, Tu HY, Murciano-Goroff Y, Xu C, Makhnin A, Martinez A, Pavlakis N, Clarke S, Itchins M, Lee A, Rimner A, Gomez D, Rocco G, Chaft JE, Riely GJ, Rudin CM, Jones DR, Li M, Shaffer T, Hosseini SA, Bertucci C, Lim LP, Drilon A, Berger MF, Benayed R, Arcila ME, Isbell JM, Li BT.	Mondaca S	Lung Cancer	2021	10.1016/j.lungcan.2021.09.016
124	First Case Report of Pregnancy on Alectinib in a Woman With Metastatic ALK-Rearranged Lung Cancer: A Case Report	Case reports, guidelines, protocol and meeting abstracts	Scarfone G, Fumagalli M, Imbimbo M, Ceruti T, Cribiù FM, Di Loreto E, D'Incalci M, Facchin F, Fontana C, Garassino MC, Peccatori FA, Persico N, Signorelli D, Zucchetti M.	Scarfone G	J Thorac Oncol	2021	10.1016/j.jtho.2021.02.005
125	Complex genetic alterations contribute to rapid disease progression in an ALK rearrangement lung adenocarcinoma patient: a case report	Case reports, guidelines, protocol and meeting abstracts	Long X, Wu H, Yang C, Li F, Zhang M, Wu X.	Long X	Transl Cancer Res	2021	10.21037/tcr-20-3473
12	Identification of Novel Alectinib-Resistant ALK Mutation G1202K with Sensitization to Lorlatinib: A Case Report and in silico Structural Modelling	Case reports, guidelines,	Yang P, Cao R, Bao H, Wu X, Yang L, Zhu D, Zhang L, Peng L, Cai Y, Zhang W, Shao Y.	Yang P	Onco Targets	2021	10.2147/OTT.S293901

6		protocol and meeting abstracts			Ther		
127	Fatal Tumour Lysis Syndrome Induced by Brigatinib in a Lung Adenocarcinoma Patient Treated With Sequential ALK Inhibitors: A Case Report	Case reports, guidelines, protocol and meeting abstracts	Wang Y, Wang T, Xue J, Jia Z, Liu X, Li B, Li J, Li X, Wang W, Bing Z, Cao L, Cao Z, Liang N.	Wang Y	Front Pharmacol	2021	10.3389/fphar.2021.809467
128	The Emerging Importance of Tumor Genomics in Operable Non-Small Cell Lung Cancer	Reviews	Lengel HB, Connolly JG, Jones GD, Caso R, Zhou J, Sanchez-Vega F, Mastrogiacomo B, Isbell JM, Li BT, Liu Y, Rekhtman N, Jones DR.	Lengel HB	Cancers (Basel)	2021	10.3390/cancers13153656
129	Anterior mediastinal large cell neuroendocrine carcinoma with elevated AFP: A case report and review	Case reports, guidelines, protocol and meeting abstracts	Komisarof J, Qiu H, Velez MJ, Mulford D.	Komisarof J	Mol Clin Oncol	2021	10.3892/mco.2020.2196
130	Drug interaction profile of TKI alectinib allows effective and safe treatment of ALK+ lung cancer in the kidney transplant recipient	Data not related or missing	Bilek O, Holanek M, Jurica J, Stepankova S, Vasina J, Selingerova I, Poprach A, Borilova S, Kazda T, Kiss I, Zdrazilova-Dubska L.	Bilek O	Int Immunopharmacol	2021	10.1016/j.intimp.2021.108012
131	An Liquid Chromatography-Tandem Mass Spectrometry Method for the Simultaneous Determination of Afatinib, Alectinib, Ceritinib, Crizotinib, Dacomitinib, Erlotinib, Gefitinib, and Osimertinib in Human Serum	Data not related or missing	Mukai Y, Wakamoto A, Hatsuyama T, Yoshida T, Sato H, Fujita A, Inotsume N, Toda T.	Mukai Y	Ther Drug Monit	2021	10.1097/FTD.0000000000000895
132	Development and validation of an HPLC-MS/MS method to simultaneously quantify alectinib, crizotinib, erlotinib, gefitinib and osimertinib in human plasma samples, using one assay run	Data not related or missing	van Veelen A, van Geel R, Schoufs R, de Beer Y, Stolk LM, Hendriks LEL, Croes S.	van Veelen A	Biomed Chromatogr	2021	10.1002/bmc.5224
133	Highly sensitive fusion detection using plasma cell-free RNA in non-small-cell lung cancers	Data not related or missing	Hasegawa N, Kohsaka S, Kurokawa K, Shinno Y, Takeda Nakamura I, Ueno T, Kojima S, Kawazu M, Suehara Y, Ishijima M, Goto Y, Kojima Y, Yonemori K, Hayashi T, Saito T, Shukuya T, Takahashi F, Takahashi K, Mano H.	Hasegawa N	Cancer Sci	2021	10.1111/cas.15084
134	Comprehensive genomic profiling of Brazilian non-small cell lung cancer patients (GBOT 0118/LACOG0418)	Data not related or missing	Mascarenhas E, Gelatti AC, Araújo LH, Baldotto C, Mathias C, Zukin M, Werutsky G, Pacheco P, Gomes R, de Castro G Jr, Cordeiro de Lima VC.	Mascarenhas E	Thorac Cancer	2021	10.1111/1759-7714.13777
135	Effect of severe renal impairment on the pharmacokinetics of brigatinib	Data not related or missing	Gupta N, Hanley MJ, Kerstein D, Tugnait M, Narasimhan N, Marbury TC, Venkatakrishnan K.	Gupta N	Invest New Drugs	2021	10.1007/s10637-021-01095-5
136	Genomic characterization and outcome evaluation of kinome fusions in lung cancer revealed novel druggable fusions	Data not related or missing	Li B, Qu H, Zhang J, Pan W, Liu M, Yan X, Huang X, He X, Lin D, Liu S, Guan R, Wu Y, Ou Q, Bao H, Xu Y, Wu X, Shao Y, Lin N.	Li B	NPJ Precis Oncol	2021	10.1038/s41698-021-00221-z
137	Resistance profiles of anaplastic lymphoma kinase tyrosine kinase inhibitors in advanced non-small-cell lung cancer: a multicenter study using targeted next-generation sequencing	Data not related or missing	Lin YT, Chiang CL, Hung JY, Lee MH, Su WC, Wu SY, Wei YF, Lee KY, Tseng YH, Su J, Chung HP, Lin CB, Ku WH, Chiang TS, Chiu CH, Shih JY.	Lin YT	Eur J Cancer	2021	10.1016/j.ejca.2021.06.043
138	Molecular profiling and utility of cell-free DNA in nonsmall carcinoma of the lung: Study in a tertiary care hospital	Data not related or missing	Ghosh M, Mukhopadhyay M, Das C, Chatterjee S, Naskar BG.	Ghosh M	J Cancer Res Ther	2021	10.4103/jcrt.JCRT_99_20
139	Treatment of Non-Small-Cell Lung Cancer Based on Circulating Cell-Free DNA and Impact of Variation Allele Frequency	Data not related or missing	Bustamante Alvarez JG, Janse S, Owen DH, Kiourtsis S, Bertino EM, He K, Carbone DP, Otterson GA.	Bustamante Alvare	Clin Lung Cancer	2021	10.1016/j.cllc.2020.11.007

				z JG			
140	Cell-Free Circulating Tumor DNA Improves Standard Genotyping of Non-Small-Cell Lung Cancer and Increases Detection of Targetable Alterations in a Selected Hispanic Cohort	Data not related or missing	Zatarain-Barrón ZL, Cardona AF, Díaz-García D, Trejo Rosales R, Rojas L, Cruz-Rico G, Nagy R, Cabrera L, Vargas C, Saam J, Barrón F, Arrieta O.	Zatarain-Barrón ZL	Oncology	2021	10.1159/000514648
141	Brigatinib Versus Crizotinib in Advanced ALK Inhibitor-Naive ALK-Positive Non-Small Cell Lung Cancer: Second Interim Analysis of the Phase III ALTA-1L Trial	Data not related or missing	Camidge DR, Kim HR, Ahn MJ, Yang JCH, Han JY, Hochmair MJ, Lee KH, Delmonte A, García Campelo MR, Kim DW, Griesinger F, Felip E, Califano R, Spira A, Gettinger SN, Tiseo M, Lin HM, Gupta N, Hanley MJ, Ni Q, Zhang P, Popat S.	Camidge DR	J Clin Oncol	2020	10.1200/JCO.20.00505
142	Frontiers of ctDNA, targeted therapies, and immunotherapy in non-small-cell lung cancer	Reviews	Zhu C, Zhuang W, Chen L, Yang W, Ou WB.	Zhu C	Transl Lung Cancer Res	2020	10.21037/tlcr.2020.01.09
143	The role of plasma genotyping in ALK- and ROS1-rearranged lung cancer	Reviews	Dagogo-Jack I, Ritterhouse LL.	Dagogo-Jack I	Transl Lung Cancer Res	2020	10.21037/tlcr-2019-cnsclc-09
144	Liquid biopsy is a valuable tool in the diagnosis and management of lung cancer	Reviews	Cecchini MJ, Yi ES.	Cecchini MJ	J Thorac Dis	2020	10.21037/jtd.2020.04.20
145	Serum CEA and CYFRA Levels in ALK-rearranged NSCLC Patients: Correlation With Distant Metastasis	Data not related or missing	Numata T, Endo T, Yanai H, Ota K, Yamamoto Y, Shimizu K, Yamada H, Hayashihara K, Okauchi S, Satoh H, Yamada Y, Tamura T, Saito K, Kikuchi N, Kurishima K, Ishikawa H, Watanabe H, Shiozawa T, Hizawa N, Funayama Y, Hayashi S, Nakamura H, Yamashita T.	Numata T	In Vivo	2020	10.21873/invivo.12013
146	Detection of ALK Gene Rearrangement in Cell-free RNA from Lung Cancer Malignant Pleural Effusion	Data not related or missing	Chu M, Zhu Y, Hu J, Zhang Z, Luo M, Ma X.	Chu M	Biomed Res Int	2020	10.1155/2020/6124106
147	Clinical utility of liquid biopsy for the diagnosis and monitoring of EML4-ALK NSCLC patients	Reviews	Sánchez-Herrero E, Provencio M, Romero A.	Sánchez-Herrero E	Adv Lab Med	2020	10.1515/almed-2019-0019
148	Correlation of plasma exosomal microRNAs with the efficacy of immunotherapy in EGFR/ALK wild-type advanced non-small cell lung cancer	Data not related or missing	Peng XX, Yu R, Wu X, Wu SY, Pi C, Chen ZH, Zhang XC, Gao CY, Shao YW, Liu L, Wu YL, Zhou Q.	Peng XX	J Immunother Cancer	2020	10.1136/jitc-2019-000376
149	MET Alterations Are a Recurring and Actionable Resistance Mechanism in ALK-Positive Lung Cancer	Data not related or missing	Dagogo-Jack I, Yoda S, Lennerz JK, Langenbucher A, Lin JJ, Rooney MM, Prutisto-Chang K, Oh A, Adams NA, Yeap BY, Chin E, Do A, Marble HD, Stevens SE, Digumarthy SR, Saxena A, Nagy RJ, Benes CH, Azzoli CG, Lawrence MS, Gainor JF, Shaw AT, Hata AN.	Dagogo-Jack I	Clin Cancer Res	2020	10.1158/1078-0432.CCR-19-3906
150	Association between circulating tumor DNA burden and disease burden in patients with ALK-positive lung cancer	Data not related or missing	Zhang EW, Dagogo-Jack I, Kuo A, Rooney MM, Shaw AT, Digumarthy SR.	Zhang EW	Cancer	2020	10.1002/cncr.33118
151	ALK Mutation Status Before and After Alectinib Treatment in Locally Advanced or Metastatic ALK-Positive NSCLC: Pooled Analysis of Two Prospective Trials	Data not related or missing	Noé J, Lovejoy A, Ou SI, Yaung SJ, Bordogna W, Klass DM, Cummings CA, Shaw AT.	Noé J	J Thorac Oncol	2020	10.1016/j.jtho.2019.10.015
152	Primary mucinous carcinomas of the lung: Clinical characteristics and treatment outcomes	Reviews	Rajeev LK, Thottian AGF, Amirtham U, Lokanatha D, Jacob LA, Babu MCS, Lokesh KN, Rudresha AH, Saldanha S, Hassan SA.	Rajeev LK	Lung India	2020	10.4103/lungindia.lungindia_52_20
153	Novel ALK mutation with durable response to brigatinib-a case report	Case reports, guidelines, protocol and meeting abstracts	Latif H, Liu SV.	Latif H	Transl Lung Cancer Res	2020	10.21037/tlcr-20-145

154	Longitudinal therapy monitoring of ALK-positive lung cancer by combined copy number and targeted mutation profiling of cell-free DNA	Data not related or missing	Dietz S, Christopoulos P, Yuan Z, Angeles AK, Gu L, Volckmar AL, Ogrodnik SJ, Janke F, Fratte CD, Zemojtel T, Schneider MA, Kazdal D, Endris V, Meister M, Muley T, Cecchin E, Reck M, Schlesner M, Thomas M, Stenzinger A, Sültmann H.	Dietz S	EBioMedicine	2020	10.1016/j.ebiom.2020.103103
155	Genomic Profiling of Circulating Tumor DNA Predicts Outcome and Demonstrates Tumor Evolution in ALK-Positive Non-Small Cell Lung Cancer Patients	Data not related or missing	Madsen AT, Winther-Larsen A, McCulloch T, Meldgaard P, Sorensen BS.	Madsen AT	Cancers (Basel)	2020	10.3390/cancers12040947
156	Responses to ALK Inhibitor Treatments in a Patient with Non-Small Cell Lung Cancer Harboring a Novel HPCAL1-ALK Fusion Variant: A Case Report	Case reports, guidelines, protocol and meeting abstracts	Wang R, Qin J, Fan Y, Li Z, Chen C, Su W.	Wang R	Onco Targets Ther	2020	10.2147/OTT.S252210
157	Clinical Relevance of an Amplicon-Based Liquid Biopsy for Detecting ALK and ROS1 Fusion and Resistance Mutations in Patients With Non-Small-Cell Lung Cancer	Data not related or missing	Mezquita L, Swalduz A, Jovelet C, Ortiz-Cuaran S, Howarth K, Planchard D, Avrillon V, Recondo G, Marteau S, Benitez JC, De Kievit F, Plagnol V, Lacroix L, Odier L, Rouleau E, Fournel P, Caramella C, Tissot C, Adam J, Woodhouse S, Nicotra C, Auclin E, Remon J, Morris C, Green E, Massard C, Pérol M, Friboulet L, Besse B, Saintigny P.	Mezquita L	JCO Precis Oncol	2020	10.1200/PO.19.00281
158	Diagnostic Accuracy of Next Generation Sequencing Panel using Circulating Tumor DNA in Patients with Advanced Non-Small Cell Lung Cancer: A Systematic Review and Meta-Analysis	Reviews	Sebastião MM, Ho RS, de Carvalho JPV, Nussbaum M.	Sebastião MM	J Health Econ Outcomes Res	2020	10.36469/jheor.2020.17088
159	Immune Checkpoint Inhibitor Therapy Achieved Complete Response for Drug-Sensitive EGFR/ALK Mutation-Negative Metastatic Pulmonary Large-Cell Neuroendocrine Carcinoma with High Tumor Mutation Burden: A Case Report	Case reports, guidelines, protocol and meeting abstracts	Zhang X, Sun Y, Miao Y, Xu S.	Zhang X	Onco Targets Ther	2020	10.2147/OTT.S259893
160	Comparison of liquid-based to tissue-based biopsy analysis by targeted next generation sequencing in advanced non-small cell lung cancer: a comprehensive systematic review	Reviews	Esagian SM, Grigoriadou GΙ, Nikas IP, Boikou V, Sadow PM, Won JK, Economopoulos KP.	Esagian SM	J Cancer Res Clin Oncol	2020	10.1007/s00432-020-03267-x
161	STRN-ALK Fusion in Lung Adenocarcinoma with Excellent Response Upon Alectinib Treatment: A Case Report and Literature Review	Reviews	Su C, Jiang Y, Jiang W, Wang H, Liu S, Shao Y, Zhao W, Ning R, Yu Q.	Su C	Onco Targets Ther	2020	10.2147/OTT.S282933
162	Durable Complete Response to Alectinib in a Lung Adenocarcinoma Patient With Brain Metastases and Low-Abundance EML4-ALK Variant in Liquid Biopsy: A Case Report	Case reports, guidelines, protocol and meeting abstracts	Zhu Y, Jia R, Shao YW, Zhu L, Ou Q, Yu M, Wu X, Zhang Y.	Zhu Y	Front Oncol	2020	10.3389/fonc.2020.01259
163	Acquired multiple mutations ALK I1171N, L1196M and G1202R mediate lorlatinib resistance in EML4-ALK-rearranged malignant pleural mesothelioma: a case report	Case reports, guidelines, protocol and meeting abstracts	Hu J, Zhang B, Yao F, Fu Y, Chen D, Li D, Du N, Lizaso A, Song J, Zhang L, Li X.	Hu J	Ther Adv Respir Dis	2020	10.1177/1753466620935770
164	Breast metastasis from EGFR/ALK negative lung adenocarcinoma: A case report	Case reports, guidelines, protocol and meeting abstracts	Cao L, Lv L.	Cao L	Medicine (Baltimore)	2020	10.1097/MD.0000000000023503
1	A proposal for score assignment to characterize biological processes from mass spectral	Reviews	Roder J, Net L, Oliveira C, Meyer K, Asmellash S, Kasimir-Bauer S, Pass H, Weber J, Roder	Roder	Clin Mass	2020	10.1016/j.clinms.2020.0

65	analysis of serum		H, Grigorieva J.	J	Spectrom		9.001
166	A case of one lung adenocarcinoma patient harboring a novel FAM179A-ALK (F1, A19) rearrangement responding to lorlatinib treatment	Case reports, guidelines, protocol and meeting abstracts	Yan J, Zhou X, Pan D.	Yan J	Lung Cancer	2020	10.1016/j.lungcan.2020.06.026
167	Early Blood-based Liquid Biopsy in Patients with Treatment-naïve Metastatic Adenocarcinoma of the Lung: A Case Series	Case reports, guidelines, protocol and meeting abstracts	Peled M, Bar J, Avni L, Chatterji S, Somech D, Dvir A, Soussan-Gutman L, Onn A.	Peled M	Isr Med Assoc J	2020	https://www.ima.org.il/MedicineIMAJ/viewarticle.aspx?year=2020&month=12&page=784
168	[Different Gene Mutation Spectrum of the Paired CSF and Plasma Samples in Lung Adenocarcinoma with Leptomeningeal Metastases: the Liquid Biopsy Based on Circulating Tumor DNA]	Case reports, guidelines, protocol and meeting abstracts	Li H, Xie Y, Lin Y, Yu T, Yin Z.	Li H	Zhongguo Fei Ai Za Zhi	2020	10.3779/j.issn.1009-3419.2020.102.14
169	Sequential ALK inhibitor treatment benefits patient with leptomeningeal metastasis harboring non-EML4-ALK rearrangements detected from cerebrospinal fluid: A case report	Case reports, guidelines, protocol and meeting abstracts	Li Z, Li P, Yan B, Gao Q, Jiang X, Zhan Z, Yan Q, Lizaso A, Huang C.	Li Z	Thorac Cancer	2020	10.1111/1759-7714.13259
170	Next-generation sequencing to dynamically detect mechanisms of resistance to ALK inhibitors in ALK-positive NSCLC patients: a case report	Case reports, guidelines, protocol and meeting abstracts	Sánchez-Herrero E, Blanco Clemente M, Calvo V, Provencio M, Romero A.	Sánchez-Herrero E	Transl Lung Cancer Res	2020	10.21037/tlcr.2020.02.07
171	Emergence of a HER2-amplified clone during disease progression in an ALK-rearranged NSCLC patient treated with ALK-inhibitors: a case report	Case reports, guidelines, protocol and meeting abstracts	Minari R, Gnetti L, Lagrasta CA, Squadrilli A, Bordi P, Azzoni C, Bottarelli L, Cosenza A, Ferri L, Caruso G, Silini EM, Tiseo M.	Minari R	Transl Lung Cancer Res	2020	10.21037/tlcr.2020.04.03
172	First case of bronchiolar adenoma lined purely by mucinous luminal cells with molecular analysis: A case report	Case reports, guidelines, protocol and meeting abstracts	Liu S, Liu N, Xiao M, Wang L, Wang EH.	Liu S	Medicine (Baltimore)	2020	10.1097/MD.0000000000022322
173	Assessment of a Highly Curated Somatic Oncology Database to Aid in the Interpretation of Clinically Important Variants in Next-Generation Sequencing Results	Data not related or missing	Yaung SJ, Krishna S, Xi L, Ju C, Palma JF, Schmid M.	Yaung SJ	J Mol Diagn	2020	10.1016/j.jmoldx.2020.08.004
174	Establishment and application of a method of next generation sequencing of 285 genes in lung cancer based on Ion-Proton platform	Data not related or missing	Chen Y, Zhang XC, Yan WQ, Guo WB, Xie Z, Lu DX, Lv ZY, Chen ZH, Su J.	Chen Y	Transl Cancer Res	2020	10.21037/tcr-19-2855
175	The Effect of Rifampin on the Pharmacokinetics and Safety of Lorlatinib: Results of a Phase One, Open-Label, Crossover Study in Healthy Participants	Data not related or missing	Chen J, Xu H, Pawlak S, James LP, Peltz G, Lee K, Ginman K, Bergeron M, Pithavala YK.	Chen J	Adv Ther	2020	10.1007/s12325-019-01198-9

176	Outcomes in oncogenic-addicted advanced NSCLC patients with actionable mutations identified by liquid biopsy genomic profiling using a tagged amplicon-based NGS assay	Data not related or missing	Remon J, Swalduz A, Planchard D, Ortiz-Cuaran S, Mezquita L, Lacroix L, Jovelet C, Rouleau E, Leonce C, De Kievit F, Morris C, Jones G, Mercier K, Howarth K, Green E, Pérol M, Saintigny P, Besse B.	Remon J	PLoS One	2020	10.1371/journal.pone.0234302
177	Identification of Deleterious NOTCH Mutation as Novel Predictor to Efficacious Immunotherapy in NSCLC	Data not related or missing	Zhang K, Hong X, Song Z, Xu Y, Li C, Wang G, Zhang Y, Zhao X, Zhao Z, Zhao J, Huang M, Huang D, Qi C, Gao C, Cai S, Gu F, Hu Y, Xu C, Wang W, Lou Z, Zhang Y, Liu L.	Zhang K	Clin Cancer Res	2020	10.1158/1078-0432.CCR-19-3976
178	Diagnostic and prognostic role of liquid biopsy in non-small cell lung cancer: evaluation of circulating biomarkers	Data not related or missing	Vicidomini G, Cascone R, Carlucci A, Fiorelli A, Di Domenico M, Santini M.	Vicidomini G	Explor Target Antitumor Ther	2020	10.37349/etat.2020.00020
179	The landscape of kinase domain duplication in Chinese lung cancer patients	Data not related or missing	Wu D, Xie Y, Jin C, Qiu J, Hou T, Du H, Chen S, Xiang J, Shi X, Liu J.	Wu D	Ann Transl Med	2020	10.21037/atm-20-7408
180	Oncogenic Genetic Alterations in Non-Small-Cell Lung Cancer (NSCLC) in Southwestern China	Data not related or missing	Ma Y, Li Q, Du Y, Chen W, Zhao G, Liu X, Li H, Liu J, Shen Z, Ma L, Zhou Y.	Ma Y	Cancer Manag Res	2020	10.2147/CMAR.S266069
181	Circulating Tumor DNA Analysis for Patients with Oncogene-Addicted NSCLC With Isolated Central Nervous System Progression	Data not related or missing	Aldea M, Hendriks L, Mezquita L, Jovelet C, Planchard D, Auclin E, Remon J, Howarth K, Benitez JC, Gazzah A, Lavaud P, Naltet C, Lacroix L, de Kievit F, Morris C, Green E, Ngo-Camus M, Rouleau E, Massard C, Caramella C, Friboulet L, Besse B.	Aldea M	J Thorac Oncol	2020	10.1016/j.jtho.2019.11.024
182	Chronic Plasma Exposure to Kinase Inhibitors in Patients with Oncogene-Addicted Non-Small Cell Lung Cancer	Data not related or missing	Geraud A, Mezquita L, Auclin E, Combarel D, Delahousse J, Gougis P, Massard C, Jovelet C, Caramella C, Adam J, Naltet C, Lavaud P, Gazzah A, Lacroix L, Rouleau E, Vasseur D, Mir O, Planchard D, Paci A, Besse B.	Geraud A	Cancers (Basel)	2020	10.3390/cancers12123758
183	Effects of Strong CYP2C8 or CYP3A Inhibition and CYP3A Induction on the Pharmacokinetics of Brigatinib, an Oral Anaplastic Lymphoma Kinase Inhibitor, in Healthy Volunteers	Data not related or missing	Tugnait M, Gupta N, Hanley MJ, Sonnichsen D, Kerstein D, Dorer DJ, Venkatakrishnan K, Narasimhan N.	Tugnait M	Clin Pharmacol Drug Dev	2020	10.1002/cpdd.723
184	Pulmonary Lymphoepithelioma-like Carcinoma	Reviews	Sathirareuangchai S, Hirata K.	Sathirareuangchai S	Arch Pathol Lab Med	2019	10.5858/arpa.2018-0149-RS
185	Liquid Biopsy and Lung Cancer	Reviews	Pisapia P, Malapelle U, Troncone G.	Pisapia P	Acta Cytol	2019	10.1159/000492710
186	Updated Efficacy and Safety Data and Impact of the EML4-ALK Fusion Variant on the Efficacy of Alectinib in Untreated ALK-Positive Advanced Non-Small Cell Lung Cancer in the Global Phase III ALEX Study	Reviews	Camidge DR, Dziadziuszko R, Peters S, Mok T, Noe J, Nowicka M, Gadgeel SM, Cheema P, Pavlakis N, de Marinis F, Cho BC, Zhang L, Moro-Sibilot D, Liu T, Bordogna W, Balas B, Müller B, Shaw AT.	Camidge DR	J Thorac Oncol	2019	10.1016/j.jtho.2019.03.007
187	ALK Resistance Mutations and Efficacy of Lorlatinib in Advanced Anaplastic Lymphoma Kinase-Positive Non-Small-Cell Lung Cancer	Data not related or missing	Shaw AT, Solomon BJ, Besse B, Bauer TM, Lin CC, Soo RA, Riely GJ, Ou SI, Clancy JS, Li S, Abbattista A, Thurm H, Satouchi M, Camidge DR, Kao S, Chiari R, Gadgeel SM, Felip E, Martini JF.	Shaw AT	J Clin Oncol	2019	10.1200/JCO.18.02236
188	Clinical Implications of Plasma-Based Genotyping With the Delivery of Personalized Therapy in Metastatic Non-Small Cell Lung Cancer	Reviews	Aggarwal C, Thompson JC, Black TA, Katz SI, Fan R, Yee SS, Chien AL, Evans TL, Bauml JM, Alley EW, Ciunci CA, Berman AT, Cohen RB, Lieberman DB, Majmundar KS, Savitch SL, Morrissette JJD, Hwang WT, Elenitoba-Johnson KSJ, Langer CJ, Carpenter EL.	Aggarwal C	JAMA Oncol	2019	10.1001/jamaoncol.2018.4305
189	How I treat ALK-positive non-small cell lung cancer	Reviews	McCusker MG, Russo A, Scilla KA, Mehra R, Rolfo C.	McCusker MG	ESMO Open	2019	10.1136/esmoopen-2019-000524
19	Circulating free tumor DNA in non-small cell lung cancer (NSCLC): clinical application and future perspectives	Reviews	Herbreteau G, Vallée A, Charpentier S, Normanno N, Hofman P, Denis MG.	Herbreteau G	J Thorac Dis	2019	10.21037/jtd.2018.12.18

0							
191	Role of liquid biopsy in oncogene-addicted non-small cell lung cancer	Reviews	Canale M, Pasini L, Bronte G, Delmonte A, Cravero P, Crinò L, Ulivi P.	Canale M	Transl Lung Cancer Res	2019	10.21037/tlcr.2019.09.15
192	Liquid biopsy tracking of lung tumor evolutions over time	Reviews	Russo A, De Miguel Perez D, Gunasekaran M, Scilla K, Lapidus R, Cooper B, Mehra R, Adamo V, Malapelle U, Rolfo C.	Russo A	Expert Rev Mol Diagn	2019	10.1080/14737159.2020.1680287
193	Treatment with Next-Generation ALK Inhibitors Fuels Plasma ALK Mutation Diversity	Data not related or missing	Dagogo-Jack I, Rooney M, Lin JJ, Nagy RJ, Yeap BY, Hubbeling H, Chin E, Ackil J, Farago AF, Hata AN, Lennerz JK, Gainor JF, Lanman RB, Shaw AT.	Dagogo-Jack I	Clin Cancer Res	2019	10.1158/1078-0432.CCR-19-1436
194	Targeted Tissue and Cell-Free Tumor DNA Sequencing of Advanced Lung Squamous-Cell Carcinoma Reveals Clinically Significant Prevalence of Actionable Alterations	Reviews	Lam VK, Tran HT, Banks KC, Lanman RB, Rinsurongkawong W, Peled N, Lewis J, Lee JJ, Roth J, Roarty EB, Swisher S, Talasaz A, Futreal PA, Papadimitrakopoulou V, Heymach JV, Zhang J.	Lam VK	Clin Lung Cancer	2019	10.1016/j.cllc.2018.08.020
195	Monitoring Therapeutic Response and Resistance: Analysis of Circulating Tumor DNA in Patients With ALK+ Lung Cancer	Data not related or missing	Horn L, Whisenant JG, Wakelee H, Reckamp KL, Qiao H, Leal TA, Du L, Hernandez J, Huang V, Blumenschein GR, Waqar SN, Patel SP, Nieva J, Oxnard GR, Sanborn RE, Shaffer T, Garg K, Holzhausen A, Harrow K, Liang C, Lim LP, Li M, Lovly CM.	Horn L	J Thorac Oncol	2019	10.1016/j.jtho.2019.08.003
196	The presence and variant allele fraction of EGFR mutations in ctDNA and development of resistance	Reviews	O'Kane GM, Liu G, Stockley TL, Shabir M, Zhang T, Law JH, Le LW, Sacher A, Shepherd FA, Bradbury PA, Leighl NB.	O'Kane GM	Lung Cancer	2019	10.1016/j.lungcan.2019.03.019
197	Clinical Utility of Cerebrospinal Fluid Cell-Free DNA as Liquid Biopsy for Leptomeningeal Metastases in ALK-Rearranged NSCLC	Reviews	Zheng MM, Li YS, Jiang BY, Tu HY, Tang WF, Yang JJ, Zhang XC, Ye JY, Yan HH, Su J, Zhou Q, Zhong WZ, Yang XN, Guo WB, Chuai S, Zhang Z, Chen HJ, Wang Z, Liu C, Wu YL.	Zheng MM	J Thorac Oncol	2019	10.1016/j.jtho.2019.01.007
198	Molecular and clinical analysis of Chinese patients with anaplastic lymphoma kinase (ALK)-rearranged non-small cell lung cancer	Reviews	Zhou X, Shou J, Sheng J, Xu C, Ren S, Cai X, Chu Q, Wang W, Zhen Q, Zhou Y, Li W, Pan H, Li H, Sun T, Cheng H, Wang H, Lou F, Rao C, Cao S, Pan H, Fang Y.	Zhou X	Cancer Sci	2019	10.1111/cas.14177
199	Molecular findings reveal possible resistance mechanisms in a patient with ALK-rearranged lung cancer: a case report and literature review	Case reports, guidelines, protocol and meeting abstracts	Kougioumtzi A, Ntellas P, Papadopoulou E, Nasioulas G, Kampletsas E, Pentheroudakis G.	Kougioumtzi A	ESMO Open	2019	10.1136/esmoopen-2019-000561
200	Clinical utility of plasma-based digital next-generation sequencing in oncogene-driven non-small-cell lung cancer patients with tyrosine kinase inhibitor resistance	Reviews	Zugazagoitia J, Gómez-Rueda A, Jantus-Lewintre E, Isla D, Camps C, Ramos I, Trigo JM, Bernabé R, Juan-Vidal O, Sanchez-Torres JM, García-Campelo R, Provencio M, Felip E, de Castro J, Faull I, Lanman RB, Ponce-Aix S, Paz-Ares L, Garrido P.	Zugazagoitia J	Lung Cancer	2019	10.1016/j.lungcan.2019.05.032
201	Quantitative detection of ALK fusion breakpoints in plasma cell-free DNA from patients with non-small cell lung cancer using PCR-based target sequencing with a tiling primer set and two-step mapping/alignment	Data not related or missing	Kunimasa K, Kato K, Imamura F, Kukita Y.	Kunimasa K	PLoS One	2019	10.1371/journal.pone.0222233
202	Oral coadministration of elacridar and ritonavir enhances brain accumulation and oral availability of the novel ALK/ROS1 inhibitor lorlatinib	Data not related or missing	Li W, Sparidans RW, Wang Y, Lebre MC, Beijnen JH, Schinkel AH.	Li W	Eur J Pharm Biopharm	2019	10.1016/j.ejpb.2019.01.016
203	Transfer of Extracellular Vesicle-Associated-RNAs Induces Drug Resistance in ALK-Translocated Lung Adenocarcinoma	Data not related or missing	Kwok HH, Ning Z, Chong PW, Wan TS, Ng MH, Ho GYF, Ip MS, Lam DC.	Kwok HH	Cancers (Basel)	2019	10.3390/cancers11010104
204	Elevated levels of pre-treatment lactate dehydrogenase are an unfavorable predictor factor in patients with EML4-ALK rearrangement non-small cell lung cancer treated with crizotinib	Data not related or missing	Liang H, Ma D, Xu Y, Zhao J, Chen M, Liu X, Zhong W, Li J, Wang M.	Liang H	Cancer Manag Res	2019	10.2147/CMAR.S213572

205	Clinicopathological characteristics with EGFR, ALK, ROS1 genetic alternation and prognostic analysis of primary lymphoepithelioma-like carcinoma	Data not related or missing	Shen Y, Hu F, Zhang B, Li C, Zhang X, Han B.	Shen Y	Transl Cancer Res	2019	10.21037/tcr.2019.09.51
206	Serial liquid biopsies for detection of treatment failure and profiling of resistance mechanisms in KLC1-ALK-rearranged lung cancer	Data not related or missing	Dietz S, Christopoulos P, Gu L, Volckmar AL, Endris V, Yuan Z, Ogrodnik SJ, Zemojtel T, Heussel CP, Schneider MA, Meister M, Muley T, Reck M, Schlesner M, Thomas M, Stenzinger A, Sültmann H.	Dietz S	Cold Spring Harb Mol Case Stud	2019	10.1101/mcs.a004630
207	Prolactin-Secreting Lung Adenocarcinoma Metastatic to the Pituitary Mimicking a Prolactinoma: A Case Report	Case reports, guidelines, protocol and meeting abstracts	Yao H, Rui W, Zhang Y, Liu Y, Lin S, Tang H, Zhao W, Wu Z.	Yao H	Neurosurgery	2019	10.1093/neuros/nyy386
208	MET Y1003S point mutation shows sensitivity to crizotinib in a patient with lung adenocarcinoma	Case reports, guidelines, protocol and meeting abstracts	Miao YL, Xu QQ.	Miao YL	Lung Cancer	2019	10.1016/j.lungcan.2019.02.002
209	Case Report: Temporal Heterogeneity of ALK Activating Mutations in Sequential ALK TKI-Treated Non-Small-Cell Lung Cancer Revealed Using NGS-Based Liquid Biopsy	Case reports, guidelines, protocol and meeting abstracts	Ding M, Deng L, Yu R, Lu D, Bai Y, Wu X, Shao YW, Yang Y.	Ding M	Clin Lung Cancer	2019	10.1016/j.cllc.2019.02.014
210	Prospective detection of mutations in cerebrospinal fluid, pleural effusion, and ascites of advanced cancer patients to guide treatment decisions	Data not related or missing	Villatoro S, Mayo-de-Las-Casas C, Jordana-Ariza N, Viteri-Ramírez S, Garzón-Ibañez M, Moya-Horno I, García-Peláez B, González-Cao M, Malapelle U, Balada-Bel A, Martínez-Bueno A, Campos R, Reguart N, Majem M, Blanco R, Blasco A, Catalán MJ, González X, Troncone G, Karachaliou N, Rosell R, Molina-Vila MA.	Villatoro S	Mol Oncol	2019	10.1002/1878-0261.12574
211	Promising preclinical platform for evaluation of immuno-oncology drugs using Hu-PBL-NSG lung cancer models	Data not related or missing	Pyo KH, Kim JH, Lee JM, Kim SE, Cho JS, Lim SM, Cho BC.	Pyo KH	Lung Cancer	2019	10.1016/j.lungcan.2018.11.035
212	A phase Ib study of the combination regorafenib with PF-03446962 in patients with refractory metastatic colorectal cancer (REGAL-1 trial)	Data not related or missing	Clarke JM, Blobe GC, Strickler JH, Uronis HE, Zafar SY, Morse M, Dropkin E, Howard L, O'Neill M, Rushing CN, Niedzwiecki D, Watson H, Bolch E, Arrowood C, Liu Y, Nixon AB, Hurwitz HI.	Clarke JM	Cancer Chemother Pharmacol	2019	10.1007/s00280-019-03916-0
213	A Low Crizotinib Concentration in the Cerebrospinal Fluid Causes Ineffective Treatment of Anaplastic Lymphoma Kinase-positive Non-small Cell Lung Cancer with Carcinomatous Meningitis	Data not related or missing	Okimoto T, Tsubata Y, Hotta T, Hamaguchi M, Nakao M, Hamaguchi SI, Hamada A, Isobe T.	Okimoto T	Intern Med	2019	10.2169/internalmedicine.1072-18
214	Different Types of ROS1 Fusion Partners Yield Comparable Efficacy to Crizotinib	Data not related or missing	He Y, Sheng W, Hu W, Lin J, Liu J, Yu B, Mao X, Zhang L, Huang J, Wang G.	He Y	Oncol Res	2019	10.3727/096504019X15509372008132
215	Identification of a novel WNK1-ROS1 fusion in a lung adenocarcinoma sensitive to crizotinib	Data not related or missing	Liu Y, Liu T, Li N, Wang T, Pu Y, Lin R.	Liu Y	Lung Cancer	2019	10.1016/j.lungcan.2018.12.011
216	Clinical utility of plasma-based digital next-generation sequencing in patients with advance-stage lung adenocarcinomas with insufficient tumor samples for tissue genotyping	Data not related or missing	Zugazagoitia J, Ramos I, Trigo JM, Palka M, Gómez-Rueda A, Jantus-Lewintre E, Camps C, Isla D, Iranzo P, Ponce-Aix S, García-Campelo R, Provencio M, Franco F, Bernabé R, Juan-Vidal O, Felip E, de Castro J, Sanchez-Torres JM, Faul I, Lanman RB, Garrido P, Paz-Ares L.	Zugazagoitia J	Ann Oncol	2019	10.1093/annonc/mdy512
2	Squamous Cell Transformation of Primary Lung Adenocarcinoma in a Patient With EML4-ALK	Data not related	Gong J, Gregg JP, Ma W, Yoneda K, Moore EH, Daly ME, Zhang Y, Williams MJ, Li T.	Gong J	J Natl	2019	10.6004/jnccn.2019.7291

17	Fusion Variant 5 Refractory to ALK Inhibitors	or missing			Compr Canc Netw		
218	Tumor-derived DNA from pleural effusion supernatant as a promising alternative to tumor tissue in genomic profiling of advanced lung cancer	Data not related or missing	Tong L, Ding N, Tong X, Li J, Zhang Y, Wang X, Xu X, Ye M, Li C, Wu X, Bao H, Zhang X, Hong Q, Song Y, Shao YW, Bai C, Zhou J, Hu J.	Tong L	Theranostics	2019	10.7150/thno.34070
219	Liquid biopsies using pleural effusion-derived exosomal DNA in advanced lung adenocarcinoma	Data not related or missing	Song Z, Cai Z, Yan J, Shao YW, Zhang Y.	Song Z	Transl Lung Cancer Res	2019	10.21037/tlcr.2019.08.14
220	Feasibility of liquid biopsy using plasma and platelets for detection of anaplastic lymphoma kinase rearrangements in non-small cell lung cancer	Data not related or missing	Park CK, Kim JE, Kim MS, Kho BG, Park HY, Kim TO, Shin HJ, Cho HJ, Choi YD, Oh IJ, Kim YC.	Park CK	J Cancer Res Clin Oncol	2019	10.1007/s00432-019-02944-w
221	Clinical implications of an analysis of pharmacokinetics of crizotinib coadministered with dexamethasone in patients with non-small cell lung cancer	Data not related or missing	Lin S, Nickens DJ, Patel M, Wilner KD, Tan W.	Lin S	Cancer Chemother Pharmacol	2019	10.1007/s00280-019-03861-y
222	Neoadjuvant Crizotinib in Resectable Locally Advanced Non-Small Cell Lung Cancer with ALK Rearrangement	Data not related or missing	Zhang C, Li SL, Nie Q, Dong S, Shao Y, Yang XN, Wu YL, Yang Y, Zhong WZ.	Zhang C	J Thorac Oncol	2019	10.1016/j.jtho.2018.10.161
223	Increased Anaplastic Lymphoma Kinase Activity Induces a Poorly Differentiated Thyroid Carcinoma in Mice	Data not related or missing	Kohler H, Latteyer S, Hönes GS, Theurer S, Liao XH, Christoph S, Zwanziger D, Schulte JH, Kero J, Undeutsch H, Refetoff S, Schmid KW, Führer D, Moeller LC.	Kohler H	Thyroid	2019	10.1089/thy.2018.0526
224	Sensitivity of next-generation sequencing assays detecting oncogenic fusions in plasma cell-free DNA	Data not related or missing	Supplee JG, Milan MSD, Lim LP, Potts KT, Sholl LM, Oxnard GR, Paweletz CP.	Supplee JG	Lung Cancer	2019	10.1016/j.lungcan.2019.06.004
225	Leptomeningeal metastasis	Reviews	Taillibert S, Chamberlain MC.	Taillibert S	Handb Clin Neurol	2018	10.1016/B978-0-12-811161-1.00013-X
226	Updated Molecular Testing Guideline for the Selection of Lung Cancer Patients for Treatment With Targeted Tyrosine Kinase Inhibitors: Guideline From the College of American Pathologists, the International Association for the Study of Lung Cancer, and the Association for Molecular Pathology	Case reports, guidelines, protocol and meeting abstracts	Lindeman NI, Cagle PT, Aisner DL, Arcila ME, Beasley MB, Bernicker EH, Colasacco C, Dacic S, Hirsch FR, Kerr K, Kwiatkowski DJ, Ladanyi M, Nowak JA, Sholl L, Temple-Smolkin R, Solomon B, Souter LH, Thunnissen E, Tsao MS, Ventura CB, Wynes MW, Yatabe Y.	Lindeman NI	Arch Pathol Lab Med	2018	10.5858/arpa.2017-0388-CP
227	Inflammatory myofibroblastic tumor of the lung	Reviews	Khatri A, Agrawal A, Sikachi RR, Mehta D, Sahni S, Meena N.	Khatri A	Adv Respir Med	2018	10.5603/ARM.2018.0007
228	Biomarker use in lung cancer management: expanding horizons	Reviews	Sandler JE, Kaumaya M, Halmos B.	Sandler JE	Biomark Med	2018	10.2217/bmm-2018-0028
229	Tissue and Blood Biomarkers in Lung Cancer: A Review	Reviews	Duffy MJ, O'Byrne K.	Duffy MJ	Adv Clin Chem	2018	10.1016/bs.acc.2018.05.001
230	Liquid Biopsy for Advanced Non-Small Cell Lung Cancer (NSCLC): A Statement Paper from the IASLC	Reviews	Rolfo C, Mack PC, Scagliotti GV, Baas P, Barlesi F, Bivona TG, Herbst RS, Mok TS, Peled N, Pirker R, Raez LE, Reck M, Riess JW, Sequist LV, Shepherd FA, Sholl LM, Tan DSW, Wakelee HA, Wistuba II, Wynes MW, Carbone DP, Hirsch FR, Gandara DR.	Rolfo C	J Thorac Oncol	2018	10.1016/j.jtho.2018.05.030
231	Therapeutic bronchoscopy in the era of genotype directed lung cancer management	Reviews	Mohan A, Harris K, Bowling MR, Brown C, Hohenforst-Schmidt W.	Mohan A	J Thorac Dis	2018	10.21037/jtd.2018.08.14

232	Circular RNA F-circEA-2a derived from EML4-ALK fusion gene promotes cell migration and invasion in non-small cell lung cancer	Data not related or missing	Tan S, Sun D, Pu W, Gou Q, Guo C, Gong Y, Li J, Wei YQ, Liu L, Zhao Y, Peng Y.	Tan S	Mol Cancer	2018	10.1186/s12943-018-0887-9
233	Anaplastic Lymphoma Kinase Mutation (ALK F1174C) in Small Cell Carcinoma of the Prostate and Molecular Response to Alectinib	Data not related or missing	Carneiro BA, Pamarthy S, Shah AN, Sagar V, Unno K, Han H, Yang XJ, Costa RB, Nagy RJ, Lanman RB, Kuzel TM, Ross JS, Gay L, Elvin JA, Ali SM, Cristofanilli M, Chae YK, Giles FJ, Abdulkadir SA.	Carneiro BA	Clin Cancer Res	2018	10.1158/1078-0432.CCR-18-0332
234	Dynamic changes of circulating tumour DNA in surgical lung cancer patients: protocol for a prospective observational study	Case reports, guidelines, protocol and meeting abstracts	Chen K, Zhao H, Yang F, Hui B, Wang T, Wang LT, Shi Y, Wang J.	Chen K	BMJ Open	2018	10.1136/bmjopen-2017-019012
235	Serum ProGRP and NSE levels predicting small cell lung cancer transformation in a patient with ALK rearrangement-positive non-small cell lung cancer: A case report	Case reports, guidelines, protocol and meeting abstracts	Oya Y, Yoshida T, Uemura T, Murakami Y, Inaba Y, Hida T.	Oya Y	Oncol Lett	2018	10.3892/ol.2018.9158
236	Baseline and On-Treatment Characteristics of Serum Tumor Markers in Stage IV Oncogene-Addicted Adenocarcinoma of the Lung	Reviews	Noonan SA, Patil T, Gao D, King GG, Thibault JR, Lu X, Bunn PA, Doebele RC, Purcell WT, Barón AE, Camidge DR.	Noonan SA	J Thorac Oncol	2018	10.1016/j.jtho.2017.08.005
237	Progressive renal insufficiency related to ALK inhibitor, alectinib	Case reports, guidelines, protocol and meeting abstracts	Nagai K, Ono H, Matsuura M, Hann M, Ueda S, Yoshimoto S, Tamaki M, Murakami T, Abe H, Ishikura H, Doi T.	Nagai K	Oxf Med Case Reports	2018	10.1093/omcr/omy009
238	Capture-based ultra-deep sequencing in plasma ctDNA reveals the resistance mechanism of ALK inhibitors in a patient with advanced ALK-positive NSCLC	Case reports, guidelines, protocol and meeting abstracts	Guo J, Guo L, Sun L, Wu Z, Ye J, Liu J, Zuo Q.	Guo J	Cancer Biol Ther	2018	10.1080/15384047.2018.1433496
239	Clinical Application of Genomic Profiling With Circulating Tumor DNA for Management of Advanced Non-Small-cell Lung Cancer in Asia	Reviews	Loong HH, Raymond VM, Shiotsu Y, Chua DTT, Teo PML, Yung T, Skrzypczak S, Lanman RB, Mok TSK.	Loong HH	Clin Lung Cancer	2018	10.1016/j.cllc.2018.04.022
240	Highly sensitive detection of ALK resistance mutations in plasma using droplet digital PCR	Data not related or missing	Yoshida R, Sasaki T, Umekage Y, Tanno S, Ono Y, Ogata M, Chiba S, Mizukami Y, Ohsaki Y.	Yoshida R	BMC Cancer	2018	10.1186/s12885-018-5031-0
241	Next generation sequencing reveals a novel ALK G1128A mutation resistant to crizotinib in an ALK-Rearranged NSCLC patient	Case reports, guidelines, protocol and meeting abstracts	Ai X, Niu X, Chang L, Chen R, Ou SI, Lu S.	Ai X	Lung Cancer	2018	10.1016/j.lungcan.2018.07.004
242	Updated Molecular Testing Guideline for the Selection of Lung Cancer Patients for Treatment With Targeted Tyrosine Kinase Inhibitors: Guideline From the College of American Pathologists, the International Association for the Study of Lung Cancer, and the Association for Molecular Pathology	Case reports, guidelines, protocol and meeting abstracts	Lindeman NI, Cagle PT, Aisner DL, Arcila ME, Beasley MB, Bernicker EH, Colasacco C, Dacic S, Hirsch FR, Kerr K, Kwiatkowski DJ, Ladanyi M, Nowak JA, Sholl L, Temple-Smolkin R, Solomon B, Souter LH, Thunnissen E, Tsao MS, Ventura CB, Wynes MW, Yatabe Y.	Lindeman NI	J Thorac Oncol	2018	10.1016/j.jtho.2017.12.001
2	5’/ 3’ imbalance strategy to detect ALK fusion genes in circulating tumor RNA from	Data not related	Tong Y, Zhao Z, Liu B, Bao A, Zheng H, Gu J, McGrath M, Xia Y, Tan B, Song C, Li Y.	Tong Y	J Exp Clin	2018	10.1186/s13046-018-0735

243	patients with non-small cell lung cancer	or missing			Cancer Res		-1
244	An UPLC-MS/MS method to determine CT-707 and its two metabolites in plasma of ALK-positive advanced non-small cell lung cancer patients	Data not related or missing	Cui C, Hu P, Jiang J, Kong F, Luo H, Zhao Q.	Cui C	J Pharm Biomed Anal	2018	10.1016/j.jpba.2018.01.042
245	[Pulmonary microcystic fibromyxoma: report of a case with review of literature]	Case reports, guidelines, protocol and meeting abstracts	Gong QX, Li H, Zhang ZH, Fan QH.	Gong QX	Zhonghua Bing Li Xue Za Zhi	2018	10.3760/cma.j.issn.0529-5807.2018.02.006
246	Therapeutic decision based on molecular detection of resistance mechanism in an ALK-rearranged lung cancer patient: a case report	Case reports, guidelines, protocol and meeting abstracts	De Carlo E, Schiappacassi M, Urbani M, Doliana R, Baldassarre G, Da Ros V, Santarossa S, Chimienti E, Berto E, Fratino L, Bearz A.	De Carlo E	Onco Targets Ther	2018	10.2147/OTT.S184745
247	Identification of a High-Level MET Amplification in CTCs and cfTNA of an ALK-Positive NSCLC Patient Developing Evasive Resistance to Crizotinib	Case reports, guidelines, protocol and meeting abstracts	Berger LA, Janning M, Velthaus JL, Ben-Batalla I, Schatz S, Falk M, Iglauer P, Simon R, Cao R, Forcato C, Manaresi N, Bramlett K, Buson G, Hanssen A, Tiemann M, Sauter G, Bokemeyer C, Riethdorf S, Reck M, Pantel K, Wikman H, Loges S.	Berger LA	J Thorac Oncol	2018	10.1016/j.jtho.2018.08.2025
248	Cauda equine syndrome as the primary symptom of leptomeningeal metastases from lung cancer: a case report and review of literature	Case reports, guidelines, protocol and meeting abstracts	Liu Y, Wang B, Qian Y, Di D, Wang M, Zhang X.	Liu Y	Onco Targets Ther	2018	10.2147/OTT.S165299
249	Possible involvement of interleukin-18 in the pathology of hepatobiliary adverse effects related to treatment with ceritinib	Data not related or missing	Hirano T, Koarai A, Ichikawa T, Sato T, Ohe T, Ichinose M.	Hirano T	BMC Cancer	2018	10.1186/s12885-018-4913-5
250	Liquid chromatography-tandem mass spectrometric assay for therapeutic drug monitoring of the EGFR inhibitors afatinib, erlotinib and osimertinib, the ALK inhibitor crizotinib and the VEGFR inhibitor nintedanib in human plasma from non-small cell lung cancer patients	Data not related or missing	Reis R, Labat L, Allard M, Boudou-Rouquette P, Chapron J, Bellesoeur A, Thomas-Schoemann A, Arrondeau J, Giraud F, Alexandre J, Vidal M, Goldwasser F, Blanchet B.	Reis R	J Pharm Biomed Anal	2018	10.1016/j.jpba.2018.05.052
251	Distinct clinicopathologic features, genomic characteristics and survival of central and peripheral pulmonary large cell neuroendocrine carcinoma: From different origin cells?	Data not related or missing	Zhou F, Hou L, Ding T, Song Q, Chen X, Su C, Li W, Gao G, Ren S, Wu F, Fan J, Wu C, Zhang J, Zhou C.	Zhou F	Lung Cancer	2018	10.1016/j.lungcan.2017.12.009
252	P-glycoprotein (MDR1/ABCB1) restricts brain accumulation and cytochrome P450-3A (CYP3A) limits oral availability of the novel ALK/ROS1 inhibitor lorlatinib	Data not related or missing	Li W, Sparidans RW, Wang Y, Lebre MC, Wagenaar E, Beijnen JH, Schinkel AH.	Li W	Int J Cancer	2018	10.1002/ijc.31582
253	Analytical validation of a next generation sequencing liquid biopsy assay for high sensitivity broad molecular profiling	Data not related or missing	Plagnol V, Woodhouse S, Howarth K, Lensing S, Smith M, Epstein M, Madi M, Smalley S, Leroy C, Hinton J, de Kievit F, Musgrave-Brown E, Herd C, Baker-Neblett K, Brennan W, Dimitrov P, Campbell N, Morris C, Rosenfeld N, Clark J, Gale D, Platt J, Calaway J, Jones G, Forshew T.	Plagnol V	PLoS One	2018	10.1371/journal.pone.0193802
254	Clinical Utility of Cell-Free DNA for the Detection of ALK Fusions and Genomic Mechanisms of ALK Inhibitor Resistance in Non-Small Cell Lung Cancer	Data not related or missing	McCoach CE, Blakely CM, Banks KC, Levy B, Chue BM, Raymond VM, Le AT, Lee CE, Diaz J, Waqar SN, Purcell WT, Aisner DL, Davies KD, Lanman RB, Shaw AT, Doebele RC.	McCoach CE	Clin Cancer Res	2018	10.1158/1078-0432.CCR-17-2588
2	Unique genetic profiles from cerebrospinal fluid cell-free DNA in leptomeningeal	Data not related	Li YS, Jiang BY, Yang JJ, Zhang XC, Zhang Z, Ye JY, Zhong WZ, Tu HY, Chen HJ, Wang Z,	Li YS	Ann Oncol	2018	10.1093/annonc/mdy009

255	metastases of EGFR-mutant non-small-cell lung cancer: a new medium of liquid biopsy	or missing	Xu CR, Wang BC, Du HJ, Chuai S, Han-Zhang H, Su J, Zhou Q, Yang XN, Guo WB, Yan HH, Liu YH, Yan LX, Huang B, Zheng MM, Wu YL.				
256	Success of Crizotinib Combined with Whole-Brain Radiotherapy for Brain Metastases in a Patient with Anaplastic Lymphoma Kinase Rearrangement-Positive Non-Small-Cell Lung Cancer	Data not related or missing	Okawa S, Shibayama T, Shimonishi A, Nishimura J, Ozeki T, Takada K, Kayatani H, Minami D, Sato K, Fujiwara K, Yonei T, Sato T, Suno M.	Okawa S	Case Rep Oncol	2018	10.1159/000492150
257	Proteolysis Targeting Chimeras (PROTACs) of Anaplastic Lymphoma Kinase (ALK)	Data not related or missing	Zhang C, Han XR, Yang X, Jiang B, Liu J, Xiong Y, Jin J.	Zhang C	Eur J Med Chem	2018	10.1016/j.ejmech.2018.03.071
258	Clinical and prognostic value of the C-Met/HGF signaling pathway in cervical cancer	Data not related or missing	Boromand N, Hasanzadeh M, ShahidSales S, Farazestanian M, Gharib M, Fiuji H, Behboodi N, Ghobadi N, Hassanian SM, Ferns GA, Avan A.	Boromand N	J Cell Physiol	2018	10.1002/jcp.26232
259	Liquid biopsy in lung cancer	Reviews	Mlika M, Hofman P, Dziri C, Mezni F.	Mlika M	Tunis Med	2017	10.1016/j.cca.2023.117757
260	Plasma genotyping in patients with non-small-cell lung cancer: simplifying or confusing the diagnosis?	Reviews	Das S, Horn L.	Das S	Lung Cancer Manag	2017	10.2217/lmt-2016-0019
261	Molecular diagnostics of lung cancer in the clinic	Reviews	Sholl L.	Sholl L	Transl Lung Cancer Res	2017	10.21037/tlcr.2017.08.03
262	Hepatocyte growth factor/MET in cancer progression and biomarker discovery	Reviews	Matsumoto K, Umitsu M, De Silva DM, Roy A, Bottaro DP.	Matsumoto K	Cancer Sci	2017	10.1111/cas.13156
263	Liquid Biopsy and Therapeutic Targets: Present and Future Issues in Thoracic Oncology	Reviews	Hofman P.	Hofman P	Cancers (Basel)	2017	10.3390/cancers9110154
264	Critical issues in the clinical application of liquid biopsy in non-small cell lung cancer	Reviews	Manicone M, Poggiana C, Facchinetti A, Zamarchi R.	Manicone M	J Thorac Dis	2017	10.21037/jtd.2017.07.28
265	What, When, and How of Biomarker Testing in Non-Small Cell Lung Cancer	Reviews	Riely GL.	Riely GL	J Natl Compr Canc Netw	2017	10.6004/jnccn.2017.0073
266	Utility of Genomic Assessment of Blood-Derived Circulating Tumor DNA (ctDNA) in Patients with Advanced Lung Adenocarcinoma	Reviews	Schwaederlé MC, Patel SP, Husain H, Ikeda M, Lanman RB, Banks KC, Talasaz A, Bazhenova L, Kurzrock R.	Schwaederlé MC	Clin Cancer Res	2017	10.1158/1078-0432.CCR-16-2497
267	Circulating microRNAs as novel biomarkers of ALK-positive nonsmall cell lung cancer and predictors of response to crizotinib therapy	Data not related or missing	Li LL, Qu LL, Fu HJ, Zheng XF, Tang CH, Li XY, Chen J, Wang WX, Yang SX, Wang L, Zhao GH, Lv PP, Zhang M, Lei YY, Qin HF, Wang H, Gao HJ, Liu XQ.	Li LL	Oncotarget	2017	10.18632/oncotarget.17535
268	A Case of Metastatic Atypical Neuroendocrine Tumor with ALK Translocation and Diffuse Brain Metastases	Data not related or missing	Wang VE, Young L, Ali S, Miller VA, Urisman A, Wolfe J, Bivona TG, Damato B, Fogh S, Bergsland EK.	Wang VE	Oncologist	2017	10.1634/theoncologist.2017-0054
269	The serum activity of thioredoxin reductases 1 (TrxR1) is correlated with the poor prognosis in EGFR wild-type and ALK negative non-small cell lung cancer	Data not related or missing	Chen G, Chen Q, Zeng F, Zeng L, Yang H, Xiong Y, Zhou C, Liu L, Jiang W, Yang N, Zhang Y.	Chen G	Oncotarget	2017	10.18632/oncotarget.23252
2	Adenocarcinoma of the lung with EGFR gene mutation and subsequent resistance mechanisms	Case reports,	Xu L, Wang QZ, Wu L.	Xu L	Onco	2017	10.2147/OTT.S143501

70	exploration: case report	guidelines, protocol and meeting abstracts			Targets Ther		
271	NGS analysis on tumor tissue and cfDNA for genotype-directed therapy in metastatic NSCLC patients. Between hope and hype?	Reviews	Falk AT, Heeke S, Hofman V, Lespinet V, Ribeyre C, Bordone O, Poudenx M, Otto J, Garnier G, Castelnau O, Guigay J, Leroy S, Marquette CH, Hofman P, Ilié M.	Falk AT	Expert Rev Anticancer Ther	2017	10.1080/14737140.2017.1331736
272	A Rare STRN-ALK Fusion in Lung Adenocarcinoma Identified Using Next-Generation Sequencing-Based Circulating Tumor DNA Profiling Exhibits Excellent Response to Crizotinib	Data not related or missing	Yang Y, Qin SK, Zhu J, Wang R, Li YM, Xie ZY, Wu Q.	Yang Y	Mayo Clin Proc Innov Qual Outcomes	2017	10.1016/j.mayocpiqo.2017.04.003
273	Emergence of novel and dominant acquired EGFR solvent-front mutations at Gly796 (G796S/R) together with C797S/R and L792F/H mutations in one EGFR (L858R/T790M) NSCLC patient who progressed on osimertinib	Case reports, guidelines, protocol and meeting abstracts	Ou SI, Cui J, Schrock AB, Goldberg ME, Zhu VW, Albacker L, Stephens PJ, Miller VA, Ali SM.	Ou SI	Lung Cancer	2017	10.1016/j.lungcan.2017.04.003
274	Dual occurrence of ALK G1202R solvent front mutation and small cell lung cancer transformation as resistance mechanisms to second generation ALK inhibitors without prior exposure to crizotinib. Pitfall of solely relying on liquid re-biopsy?	Data not related or missing	Ou SI, Lee TK, Young L, Fernandez-Rocha MY, Pavlick D, Schrock AB, Zhu VW, Milliken J, Ali SM, Gitlitz BJ.	Ou SI	Lung Cancer	2017	10.1016/j.lungcan.2017.02.005
275	Emergence of FGFR3-TACC3 fusions as a potential by-pass resistance mechanism to EGFR tyrosine kinase inhibitors in EGFR mutated NSCLC patients	Data not related or missing	Ou SI, Horn L, Cruz M, Vafai D, Lovly CM, Spradlin A, Williamson MJ, Dagogo-Jack I, Johnson A, Miller VA, Gadgeel S, Ali SM, Schrock AB.	Ou SI	Lung Cancer	2017	10.1016/j.lungcan.2017.07.006
276	Effect of alectinib on cardiac electrophysiology: results from intensive electrocardiogram monitoring from the pivotal phase II NP28761 and NP28673 studies	Data not related or missing	Morcos PN, Bogman K, Hubeaux S, Sturm-Pellanda C, Ruf T, Bordogna W, Golding S, Zeaiter A, Abt M, Balas B.	Morcos PN	Cancer Chemother Pharmacol	2017	10.1007/s00280-017-3253-5
277	Comparison of clinical and radiological characteristics between anaplastic lymphoma kinase rearrangement and epidermal growth factor receptor mutation in treatment naïve advanced lung adenocarcinoma	Data not related or missing	Miao Y, Zhu S, Li H, Zou J, Zhu Q, Lv T, Song Y.	Miao Y	J Thorac Dis	2017	10.21037/jtd.2017.08.134
278	An UPLC-MS/MS method for the quantitation of alectinib in rat plasma	Data not related or missing	Huang XX, Li YX, Li XY, Hu XX, Tang PF, Hu GX.	Huang XX	J Pharm Biomed Anal	2017	10.1016/j.jpba.2016.10.010
279	Discovery of targetable genetic alterations in advanced non-small cell lung cancer using a next-generation sequencing-based circulating tumor DNA assay	Data not related or missing	Hou H, Yang X, Zhang J, Zhang Z, Xu X, Zhang X, Zhang C, Liu D, Yan W, Zhou N, Zhu H, Qian Z, Li Z, Zhang X.	Hou H	Sci Rep	2017	10.1038/s41598-017-14962-0
280	Circulating cell-free nucleic acids and platelets as a liquid biopsy in the provision of personalized therapy for lung cancer patients	Data not related or missing	Sorber L, Zwaenepoel K, Deschoolmeester V, Van Schil PE, Van Meerbeeck J, Lardon F, Rolfo C, Pauwels P.	Sorber L	Lung Cancer	2017	10.1016/j.lungcan.2016.04.026
281	The renal effects of ALK inhibitors	Reviews	Izzedine H, El-Fekih RK, Perazella MA.	Izzedine H	Invest New Drugs	2016	10.1007/s10637-016-0379-y
282	Feasibility of cell-free circulating tumor DNA testing for lung cancer	Reviews	Santarpia M, Karachaliou N, González-Cao M, Altavilla G, Giovannetti E, Rosell R.	Santarpia M	Biomark Med	2016	10.2217/bmm.16.6
283	Identification of I1171N resistance mutation in ALK-positive non-small-cell lung cancer tumor sample and circulating tumor DNA	Case reports, guidelines, protocol and	Johnson AC, Dô P, Richard N, Dubos C, Michels JJ, Bonneau J, Gervais R.	Johnson AC	Lung Cancer	2016	10.1016/j.lungcan.2016.06.010

		meeting abstracts					
284	Alectinib's activity against CNS metastases from ALK-positive non-small cell lung cancer: a single institution case series	Case reports, guidelines, protocol and meeting abstracts	Metro G, Lunardi G, Bennati C, Chiarini P, Sperduti I, Ricciuti B, Marcomigni L, Costa C, Crinò L, Floridi P, Gori S, Chiari R.	Metro G	J Neurooncol	2016	10.1007/s11060-016-2184-z
285	Successful Chemo-Radiotherapy for Primary Anaplastic Large Cell Lymphoma of the Lung: A Case Report and Literature Review	Reviews	Zhao Q, Liu Y, Chen H, Zhang Y, Du Z, Wang J, Wang Y.	Zhao Q	Am J Case Rep	2016	10.12659/ajcr.896096
286	First-in-human, open-label dose-escalation and dose-expansion study of the safety, pharmacokinetics, and antitumor effects of an oral ALK inhibitor ASP3026 in patients with advanced solid tumors	Data not related or missing	Li T, LoRusso P, Maitland ML, Ou SH, Bahceci E, Ball HA, Park JW, Yuen G, Tolcher A.	Li T	J Hematol Oncol	2016	10.1186/s13045-016-0254-5
287	Metastatic EML4-ALK fusion detected by circulating DNA genotyping in an EGFR-mutated NSCLC patient and successful management by adding ALK inhibitors: a case report	Case reports, guidelines, protocol and meeting abstracts	Liang W, He Q, Chen Y, Chuai S, Yin W, Wang W, Peng G, Zhou C, He J.	Liang W	BMC Cancer	2016	10.1186/s12885-016-2088-5
288	Histopathological transformation to small-cell lung carcinoma in non-small cell lung carcinoma tumors	Reviews	Dorantes-Heredia R, Ruiz-Morales JM, Cano-García F.	Dorantes-Heredia R	Transl Lung Cancer Res	2016	10.21037/tlcr.2016.07.10
289	Patients harboring EGFR mutation after primary resistance to crizotinib and response to EGFR-tyrosine kinase inhibitor	Data not related or missing	Wang W, Jiang X, Song Z, Zhang Y.	Wang W	Onco Targets Ther	2016	10.2147/OTT.S97100
290	Pharmacokinetic profiles of significant adverse events with crizotinib in Japanese patients with ABCB1 polymorphism	Data not related or missing	Fujiwara Y, Hamada A, Mizugaki H, Aikawa H, Hata T, Horinouchi H, Kanda S, Goto Y, Itahashi K, Nokihara H, Yamamoto N, Ohe Y.	Fujiwara Y	Cancer Sci	2016	10.1111/cas.12983
291	MicroRNA 25, microRNA 145, and microRNA 210 as biomarkers for predicting the efficacy of maintenance treatment with pemetrexed in lung adenocarcinoma patients who are negative for epidermal growth factor receptor mutations or anaplastic lymphoma kinase translocations	Data not related or missing	Shi SB, Wang M, Tian J, Li R, Chang CX, Qi JL.	Shi SB	Transl Res	2016	10.1016/j.trsl.2015.11.006
292	Detection of Echinoderm Microtubule Associated Protein Like 4-Anaplastic Lymphoma Kinase Fusion Genes in Non-small Cell Lung Cancer Clinical Samples by a Real-time Quantitative Reverse Transcription Polymerase Chain Reaction Method	Data not related or missing	Zhao J, Zhao JY, Chen ZX, Zhong W, Li LY, Liu LC, Hu XX, Chen WJ, Wang MZ.	Zhao J	Zhongguo Yi Xue Ke Xue Yuan Xue Bao	2016	10.3881/j.issn.1000-503X.2016.06.004
293	FDG-PET in the evaluation of response to nivolumab in recurrent non-small-cell lung cancer	Data not related or missing	Higuchi M, Owada Y, Inoue T, Watanabe Y, Yamaura T, Fukuhara M, Hasegawa T, Suzuki H.	Higuchi M	World J Surg Oncol	2016	10.1186/s12957-016-0998-y
294	Evaluation of plasma microRNA levels to predict insensitivity of patients with advanced lung adenocarcinomas to pemetrexed and platinum	Data not related or missing	Zhu J, Qi Y, Wu J, Shi M, Feng J, Chen L.	Zhu J	Oncol Lett	2016	10.3892/ol.2016.5295
295	Fusion gene and splice variant analyses in liquid biopsies of lung cancer patients	Data not related or missing	Aguado C, Giménez-Capitán A, Karachaliou N, Pérez-Rosado A, Viteri S, Morales-Espinosa D, Rosell R.	Aguado C	Transl Lung Cancer Res	2016	10.21037/tlcr.2016.09.02
29	Bioanalysis of alectinib and metabolite M4 in human plasma, cross-validation and impact on PK assessment	Data not related or missing	Heinig K, Miya K, Kamei T, Guerini E, Fraier D, Yu L, Bansal S, Morcos PN.	Heinig K	Bioanalysis	2016	10.4155/bio-2016-0068

6							
297	Role of circulating-tumor DNA analysis in non-small cell lung cancer	Reviews	Jiang T, Ren S, Zhou C.	Jiang T	Lung Cancer	2015	10.1016/j.lungcan.2015.09.013
298	Focused molecular analysis of small cell lung cancer: feasibility in routine clinical practice	Reviews	Abdelraouf F, Sharp A, Maurya M, Mair D, Wotherspoon A, Leary A, Gonzalez de Castro D, Bhosle J, Nassef A, Gaafar T, Popat S, Yap TA, O'Brien M.	Abdelraouf F	BMC Res Notes	2015	10.1186/s13104-015-1675-x
299	Interstitial lung disease induced by alectinib (CH5424802/RO5424802)	Reviews	Ikeda S, Yoshioka H, Arita M, Sakai T, Sone N, Nishiyama A, Niwa T, Hotta M, Tanaka T, Ishida T.	Ikeda S	Jpn J Clin Oncol	2015	10.1093/jjco/hyu183
300	Patient-derived xenografts from non-small cell lung cancer brain metastases are valuable translational platforms for the development of personalized targeted therapy	Reviews	Lee HW, Lee JI, Lee SJ, Cho HJ, Song HJ, Jeong DE, Seo YJ, Shin S, Joung JG, Kwon YJ, Choi YL, Park WY, Lee HM, Seol HJ, Shim YM, Joo KM, Nam DH.	Lee HW	Clin Cancer Res	2015	10.1158/1078-0432.CCR-14-1589
301	Alternate-day Treatment with Crizotinib for Drug-induced Esophagitis and Liver Damage in a Patient with EML4-ALK Fusion Gene-positive Lung Adenocarcinoma	Data not related or missing	Tsukita Y, Fukuhara T, Kobayashi M, Morita M, Suzuki A, Watanabe K, Noguchi T, Kurata Y, Suno M, Maemondo M.	Tsukita Y	Intern Med	2015	10.2169/internalmedicine.54.4996
302	Evaluation of crizotinib absolute bioavailability, the bioequivalence of three oral formulations, and the effect of food on crizotinib pharmacokinetics in healthy subjects	Data not related or missing	Xu H, O'Gorman M, Boutros T, Brega N, Kantaridis C, Tan W, Bello A.	Xu H	J Clin Pharmacol	2015	10.1002/jcph.356
303	Crizotinib-induced pancreatic pseudocyst: a novel adverse event	Data not related or missing	Ishida H, Ichikawa W, Sasaki Y.	Ishida H	BMJ Case Rep	2015	10.1136/bcr-2015-211556
304	P-glycoprotein Mediates Ceritinib Resistance in Anaplastic Lymphoma Kinase-rearranged Non-small Cell Lung Cancer	Data not related or missing	Katayama R, Sakashita T, Yanagitani N, Ninomiya H, Horiike A, Friboulet L, Gainor JF, Motoi N, Dobashi A, Sakata S, Tambo Y, Kitazono S, Sato S, Koike S, John Iafrate A, Mino-Kenudson M, Ishikawa Y, Shaw AT, Engelman JA, Takeuchi K, Nishio M, Fujita N.	Katayama R	EBioMedicine	2015	10.1016/j.ebiom.2015.12.009
305	Molecular profiling of small cell lung cancer in a Japanese cohort	Case reports, guidelines, protocol and meeting abstracts	Wakuda K, Kenmotsu H, Serizawa M, Koh Y, Isaka M, Takahashi S, Ono A, Taira T, Naito T, Murakami H, Mori K, Endo M, Nakajima T, Ohde Y, Takahashi T, Yamamoto N.	Wakuda K	Lung Cancer	2014	10.1016/j.lungcan.2014.02.013
306	Fulminant hepatitis following crizotinib administration for ALK-positive non-small-cell lung carcinoma	Case reports, guidelines, protocol and meeting abstracts	Sato Y, Fujimoto D, Shibata Y, Seo R, Suginoshita Y, Imai Y, Tomii K.	Sato Y	Jpn J Clin Oncol	2014	10.1093/jjco/hyu086
307	Serum carcinoembryonic antigen levels before initial treatment are associated with EGFR mutations and EML4- ALK fusion gene in lung adenocarcinoma patients	Data not related or missing	Wang WT, Li Y, Ma J, Chen XB, Qin JJ.	Wang WT	Asian Pac J Cancer Prev	2014	10.7314/apjcp.2014.15.9.3927
308	Increased oral availability and brain accumulation of the ALK inhibitor crizotinib by coadministration of the P-glycoprotein (ABCB1) and breast cancer resistance protein (ABCG2) inhibitor elacridar	Data not related or missing	Tang SC, Nguyen LN, Sparidans RW, Wagenaar E, Beijnen JH, Schinkel AH.	Tang SC	Int J Cancer	2014	10.1002/ijc.28475
309	[Companion diagnostics in the era of personalized medicine--chairmen's introductory remarks]	Reviews	Fukutsuka K, Takubo T.	Fukutsuka K	Rinsho Byori	2014	https://pubmed.ncbi.nlm.nih.gov/25022067/
31	Clinical validation of an ultra high-throughput spiral microfluidics for the detection and enrichment of viable circulating tumor cells	Data not related or missing	Khoo BL, Warkiani ME, Tan DS, Bhagat AA, Irwin D, Lau DP, Lim AS, Lim KH, Krisna SS, Lim WT, Yap YS, Lee SC, Soo RA, Han J, Lim CT.	Khoo BL	PLoS One	2014	10.1371/journal.pone.0099409

0							
311	The selective anaplastic lymphoma receptor tyrosine kinase inhibitor ASP3026 induces tumor regression and prolongs survival in non-small cell lung cancer model mice	Data not related or missing	Mori M, Ueno Y, Konagai S, Fushiki H, Shimada I, Kondoh Y, Saito R, Mori K, Shindou N, Soga T, Sakagami H, Furutani T, Doihara H, Kudoh M, Kuromitsu S.	Mori M	Mol Cancer Ther	2014	10.1158/1535-7163.MCT-13-0395
312	Antitumor activity of the selective ALK inhibitor alectinib in models of intracranial metastases	Data not related or missing	Kodama T, Hasegawa M, Takanashi K, Sakurai Y, Kondoh O, Sakamoto H.	Kodama T	Cancer Chemother Pharmacol	2014	10.1007/s00280-014-2578-6
313	Pulmonary inflammatory myofibroblastic tumor and IgG4-related inflammatory pseudotumor: a diagnostic dilemma	Reviews	Bhagat P, Bal A, Das A, Singh N, Singh H.	Bhagat P	Virchows Arch	2013	10.1007/s00428-013-1493-2
314	Acute kidney injury following crizotinib administration for non-small-cell lung carcinoma	Data not related or missing	Gastaud L, Ambrosetti D, Otto J, Marquette CH, Coutts M, Hofman P, Esnault V, Favre G.	Gastaud L	Lung Cancer	2013	10.1016/j.lungcan.2013.08.007
315	Inflammatory myofibroblastic tumor of the lung in children: anaplastic lymphoma kinase (ALK) expression and clinico-pathological correlation	Data not related or missing	Siminovich M, Galluzzo L, López J, Lubieniecki F, de Dávila MT.	Siminovich M	Pediatr Dev Pathol	2012	10.2350/11-10-1105-OA.1
316	Activity and safety of crizotinib in patients with ALK-positive non-small-cell lung cancer: updated results from a phase 1 study	Data not related or missing	Camidge DR, Bang YJ, Kwak EL, Iafrate AJ, Varella-Garcia M, Fox SB, Riely GJ, Solomon B, Ou SH, Kim DW, Salgia R, Fidias P, Engelman JA, Gandhi L, Jänne PA, Costa DB, Shapiro GI, Lorusso P, Ruffner K, Stephenson P, Tang Y, Wilner K, Clark JW, Shaw AT.	Camidge DR	Lancet Oncol	2012	10.1016/S1470-2045(12)70344-3
317	Clinicoradiologic characteristics of patients with lung adenocarcinoma harboring EML4-ALK fusion oncogene	Data not related or missing	Fukui T, Yatabe Y, Kobayashi Y, Tomizawa K, Ito S, Hatooka S, Matsuo K, Mitsudomi T.	Fukui T	Lung Cancer	2012	10.1016/j.lungcan.2012.03.013
318	Pharmacokinetic/pharmacodynamic modeling of crizotinib for anaplastic lymphoma kinase inhibition and antitumor efficacy in human tumor xenograft mouse models	Data not related or missing	Yamazaki S, Vicini P, Shen Z, Zou HY, Lee J, Li Q, Christensen JG, Smith BJ, Shetty B.	Yamazaki S	J Pharmacol Exp Ther	2012	10.1124/jpet.111.188870
319	Inflammatory myofibroblastic tumor of the lung with unique histological pattern and association with Sjögren's disease and systemic lupus erythematosus	Data not related or missing	Shlopov BV, French SW.	Shlopov BV	Exp Mol Pathol	2011	10.1016/j.yexmp.2011.05.008
320	Cytology of fine-needle aspiration of inflammatory myofibroblastic tumor	Data not related or missing	Stoll LM, Li QK.	Stoll LM	Diagn Cytopathol	2011	10.1002/dc.21444
321	A case report of inflammatory pseudotumor of the lung: rapid recurrence appearing as multiple lung nodules	Case reports, guidelines, protocol and meeting abstracts	Kato S, Kondo K, Teramoto T, Harada T, Ikeda H, Hara K, Nagata Y.	Kato S	Ann Thorac Cardiovasc Surg	2002	https://pubmed.ncbi.nlm.nih.gov/12472387/

Included articles

	Title	Authors	First Author	Journal	Publication Year	DOI/URL
1	Plasma ctDNA increases tissue NGS-based detection of therapeutically targetable mutations in lung cancers	Xie J, Yao W, Chen L, Zhu W, Liu Q, Geng G, Fang J, Zhao Y, Xiao L, Huang Z, Zhao J.	Xie J	BMC Cancer	2023	10.1186/s12885-023-10674-z
2	Gene alternation of cerebrospinal fluid in patients with leptomeningeal metastases of lung adenocarcinoma	Yang H, Wen L, Pan Y, Shan C, Hong W, Wang H, Zhou C, Cai L, Zhou C.	Yang H	BMC Cancer	2022	10.1186/s12885-022-0959

	using next-generation sequencing					7-y
3	Clinical Utility of Comprehensive Cell-free DNA Analysis to Identify Genomic Biomarkers in Patients with Newly Diagnosed Metastatic Non-small Cell Lung Cancer	Natasha B Leighl, Ray D Page, Victoria M Raymond, Davey B Daniel, Stephen G Divers, Karen L Reckamp, Miguel A Villalona-Calero, Daniel Dix, Justin I Odegaard, Richard B Lanman, Vassiliki A Papadimitrakopoulou	Natasha B Leighl	Clin Cancer Res	2019	10.1158/1078-0432.CCR-19-0624
4	Amplicon-based next-generation sequencing of plasma cell-free DNA for detection of driver and resistance mutations in advanced non-small cell lung cancer	Guibert N, Hu Y, Feeney N, Kuang Y, Plagnol V, Jones G, Howarth K, Beeler JF, Paweletz CP, Oxnard GR.	Guibert N	Ann Oncol	2018	10.1093/annonc/mdy005
5	Use of capture-based next-generation sequencing to detect ALK fusion in plasma cell-free DNA of patients with non-small-cell lung cancer	Cui S, Zhang W, Xiong L, Pan F, Niu Y, Chu T, Wang H, Zhao Y, Jiang L.	Cui S	Oncotarget	2017	10.18632/oncotarget.13741
6	Application of Single-Molecule Amplification and Resequencing Technology for Broad Surveillance of Plasma Mutations in Patients with Advanced Lung Adenocarcinoma	Wang Z, Cheng G, Han X, Mu X, Zhang Y, Cui D, Liu C, Zhang L, Fan Z, Ma L, Yang L, Di J, Cram DS, Shi Y, Liu D.	Wang Z	J Mol Diagn	2017	10.1016/j.jmoldx.2016.09.008
7	Development and Clinical Utility of a Blood-Based Test Service for the Rapid Identification of Actionable Mutations in Non-Small Cell Lung Carcinoma	Mellert H, Foreman T, Jackson L, Maar D, Thurston S, Koch K, et al.	Mellert H	J Mol Diagn	2017	10.1016/j.jmoldx.2016.11.004
8	Detection of circulating tumor DNA in patients with advanced non-small cell lung cancer	Yao Y, Liu J, Li L, Yuan Y, Nan K, Wu X, et al.	Yao Y	Oncotarget	2017	10.18632/oncotarget.12883
9	Noninvasive genotyping and monitoring of anaplastic lymphoma kinase (ALK) rearranged non-small cell lung cancer by capture-based next-generation sequencing	Wang Y, Tian PW, Wang WY, Wang K, Zhang Z, Chen BJ, He YQ, Li L, Liu H, Chuai S, Li WM.	Wang Y	Oncotarget	2016	10.18632/oncotarget.11569
10	Bias-Corrected Targeted Next-Generation Sequencing for Rapid, Multiplexed Detection of Actionable Alterations in Cell-Free DNA from Advanced Lung Cancer Patients	Paweletz CP, Sacher AG, Raymond CK, Alden RS, O'Connell A, Mach SL, Kuang Y, Gandhi L, Kirschmeier P, English JM, Lim LP, Jänne PA, Oxnard GR.	Paweletz CP	Clin Cancer Res	2016	10.1158/1078-0432.CCR-15-1627-T
11	Low Input Whole-Exome Sequencing to Determine the Representation of the Tumor Exome in Circulating DNA of Non-Small Cell Lung Cancer Patients	Dietz S, Schirmer U, Mercé C, von Bubnoff N, Dahl E, Meister M, Muley T, Thomas M, Sültmann H.	Dietz S	PLoS One	2016	10.1371/journal.pone.0161012
12	Rearranged EML4-ALK fusion transcripts sequester in circulating blood platelets and enable blood-based crizotinib response monitoring in non-small-cell lung cancer	Nilsson RJ, Karachaliou N, Berenguer J, Gimenez-Capitan A, Schellen P, Teixido C, et al.	Nilsson RJ	Oncotarget	2016	10.18632/oncotarget.6279
13	Detection of Therapeutically Targetable Driver and Resistance Mutations in Lung Cancer Patients by Next-Generation Sequencing of Cell-Free Circulating Tumor DNA	Thompson JC, Yee SS, Troxel AB, Savitch SL, Fan R, Balli D, et al.	Thompson JC	Clin Cancer Res	2016	10.1158/1078-0432.CCR-16-1231
