# Supplementary material for: Diagnostic accuracy of circulating tumor DNA for detection of ALK rearrangement in lung cancer: A systematic review and meta-analysis of 14 studies
Source: PLoS One. 2025 Aug 25;20(8):e0330855. doi: 10.1371/journal.pone.0330855 (PMC12377591; doi:10.1371/journal.pone.0330855)
Supplement: S2 Table — (S2 Table.PDF) [file pone.0330855.s003.pdf]

Supplementary Table 2. Characteristics of eligible studies.

Author	Year	No.of TP	No.of FP	No.of FN	No.of TN	Names of data extractors	Date of data extraction	Confirmation from a third researcher
N Guibert	2018	6	0	1	2	Lan Yang, Jiantong Sun	04/01/2024	/
Z Wang	2017	3	0	3	97	Lan Yang, Jiantong Sun	04/01/2024	/
SH Cui	2017	13	0	11	15	Lan Yang, Jiantong Sun	04/01/2024	/
Y Yao	2017	3	0	1	35	Lan Yang, Jiantong Sun	04/01/2024	Lei Li
H Mellert	2017	10	0	5	9	Lan Yang, Jiantong Sun	04/01/2024	/
CP Paweletz	2016	2	0	1	46	Lan Yang, Jiantong Sun	04/01/2024	/
S Dietz	2016	1	0	0	5	Lan Yang, Jiantong Sun	04/01/2024	/
JC Thompson	2016	1	1	0	48	Lan Yang, Jiantong Sun	04/01/2024	/
RJA Nilsson 1	2016	3	0	11	18	Lan Yang, Jiantong Sun	04/01/2024	Lei Li
RJA Nilsson 2	2016	22	0	12	33	Lan Yang, Jiantong Sun	04/01/2024	Lei Li
Y Wang	2016	19	0	5	36	Lan Yang, Jiantong Sun	04/01/2024	/
Natasha B. Leighl	2019	6	0	2	207	Lan Yang, Jiantong Sun	04/01/2024	/
H Yang	2022	1	0	1	20	Lan Yang, Jiantong Sun	04/01/2024	Lei Li
Jianjiang Xie	2023	3	5	6	409	Lan Yang, Jiantong Sun	04/01/2024	/
Natasha B. Leighl	2019	6	0	2	207	Lan Yang, Jiantong Sun	04/01/2024	/
H Yang	2022	1	0	1	20	Lan Yang, Jiantong Sun	04/01/2024	/
Jianjiang Xie	2023	3	5	6	409	Lan Yang, Jiantong Sun	04/01/2024	/

N Guibert	2018	6	0	1	2	Lan Yang, Jiantong Sun	04/01/2024	/
Z Wang	2017	3	0	3	97	Lan Yang, Jiantong Sun	04/01/2024	/
SH Cui	2017	13	0	11	15	Lan Yang, Jiantong Sun	04/01/2024	/
Y Yao	2017	3	0	1	35	Lan Yang, Jiantong Sun	04/01/2024	/
H Mellert	2017	10	0	5	9	Lan Yang, Jiantong Sun	04/01/2024	/
CP Paweletz	2016	2	0	1	46	Lan Yang, Jiantong Sun	04/01/2024	/
S Dietz	2016	1	0	0	5	Lan Yang, Jiantong Sun	04/01/2024	/
JC Thompson	2016	1	1	0	48	Lan Yang, Jiantong Sun	04/01/2024	/
RJA Nilsson 1	2016	3	0	11	18	Lan Yang, Jiantong Sun	04/01/2024	Lei Li
RJA Nilsson 2	2016	22	0	12	33	Lan Yang, Jiantong Sun	04/01/2024	Lei Li
Y Wang	2016	19	0	5	36	Lan Yang, Jiantong Sun	04/01/2024	/

Abbreviations: TP, true-positive; FP, false-positive; FN, false-negative; TN, true-negative; NA, not available (missing).
