# Supplementary material for: Diagnostic accuracy of circulating tumor DNA for detection of ALK rearrangement in lung cancer: A systematic review and meta-analysis of 14 studies
Source: PLoS One. 2025 Aug 25;20(8):e0330855. doi: 10.1371/journal.pone.0330855 (PMC12377591; doi:10.1371/journal.pone.0330855)
Supplement: S3 Table — (S3 Table.PDF) [file pone.0330855.s004.pdf]

Supplementary Table 3. QUADAS-2 tool assessment results.

	CP Paweletz 2016	H Mellert 2017	H Yang 2022	JC Thompson 2016	J Xie 2023	Natasha B 2019	N Guibert 2018	RJA Nilsson 2016	S Dietz 2016	SH Cui 2017	Y Wang 2016	Y Yao 2017	Z Wang 2017
DOMAIN 1: PATIENT SELECTION													
**A. Risk of Bias**	?	?	?	L	L	L	?	?	?	L	L	L	L
**Could the selection of patients have introduced bias?**	?	?	?	L	L	L	?	?	?	L	L	L	L
Was a consecutive or random sample of patients enrolled?	?	Y	?	Y	Y	Y	?	?	?	Y	Y	Y	Y
Was a case-control design avoided?	?	?	?	Y	Y	Y	?	Y	?	Y	Y	Y	Y
Did the study avoid inappropriate exclusions?	Y	?	?	Y	Y	Y	Y	Y	Y	Y	Y	Y	Y
**B. Concerns regarding applicability**	L	L	L	L	L	L	L	L	L	L	L	L	L
**Is there concern that the included patients do not match the review question?**	/	/	/	/	/	/	/	/	/	/	/	/	/
DOMAIN 2: INDEX TEST(S)													
**A. Risk of Bias**	L	L	L	L	L	L	L	L	L	L	H	L	L
**Could the conduct or interpretation of the index test have introduced bias?**	L	L	L	L	L	L	L	L	L	L	H	L	L
Were the index test results interpreted without knowledge of the results of the reference standard?	Y	Y	Y	Y	Y	Y	Y	Y	Y	Y	N	Y	Y
If a threshold was used, was it pre-specified?	Y	Y	Y	Y	Y	Y	Y	Y	Y	Y	Y	Y	Y
**B. Concerns regarding applicability**	L	L	L	L	L	L	L	L	L	L	L	L	L
**Is there concern that the included patients do not match the review question?**	/	/	/	/	/	/	/	/	/	/	/	/	/
DOMAIN 3: REFERENCE STANDARD													
**A. Risk of Bias**	L	L	L	L	L	?	L	L	L	L	L	L	L
**Could the reference standard, its conduct, or its interpretation have introduced bias?**	L	L	L	L	L	?	L	L	L	L	L	L	L
Is the reference standard likely to correctly classify the target condition?	Y	Y	Y	Y	Y	Y	Y	Y	Y	Y	Y	Y	Y
Were the reference standard results interpreted without knowledge of the results of the index test?	Y	Y	Y	Y	Y	?	Y	Y	Y	Y	Y	Y	Y
**B. Concerns regarding applicability**	L	L	L	L	L	L	L	L	L	L	L	L	L
**Is there concern that the included patients do not match the review question?**	/	/	/	/	/	/	/	/	/	/	/	/	/
DOMAIN 4: FLOW AND TIMING													
**A. Risk of Bias**	L	L	L	L	L	L	L	L	L	L	L	L	L
Could the patient flow have introduced bias?	Y	Y	Y	Y	Y	Y	Y	Y	Y	Y	Y	Y	Y
Was there an appropriate interval between index test(s) and reference standard?	Y	Y	Y	Y	Y	Y	Y	Y	Y	Y	Y	Y	Y
Did all patients receive a reference standard?	Y	Y	Y	Y	Y	Y	Y	Y	Y	Y	Y	Y	Y
Did patients receive the same reference standard?	Y	Y	Y	Y	Y	Y	Y	Y	Y	Y	Y	Y	Y
Were all patients included in the analysis?	Y	Y	Y	Y	Y	Y	Y	Y	Y	Y	Y	Y	Y

Abbreviations: Y,Yes; N, No; H, High; L, Low; ?, unknown.
