# Supplementary figures and images for: Diagnostic accuracy of circulating tumor DNA for detection of ALK rearrangement in lung cancer: A systematic review and meta-analysis of 14 studies
Source: PLoS One. 2025 Aug 25;20(8):e0330855. doi: 10.1371/journal.pone.0330855 (PMC12377591; doi:10.1371/journal.pone.0330855)

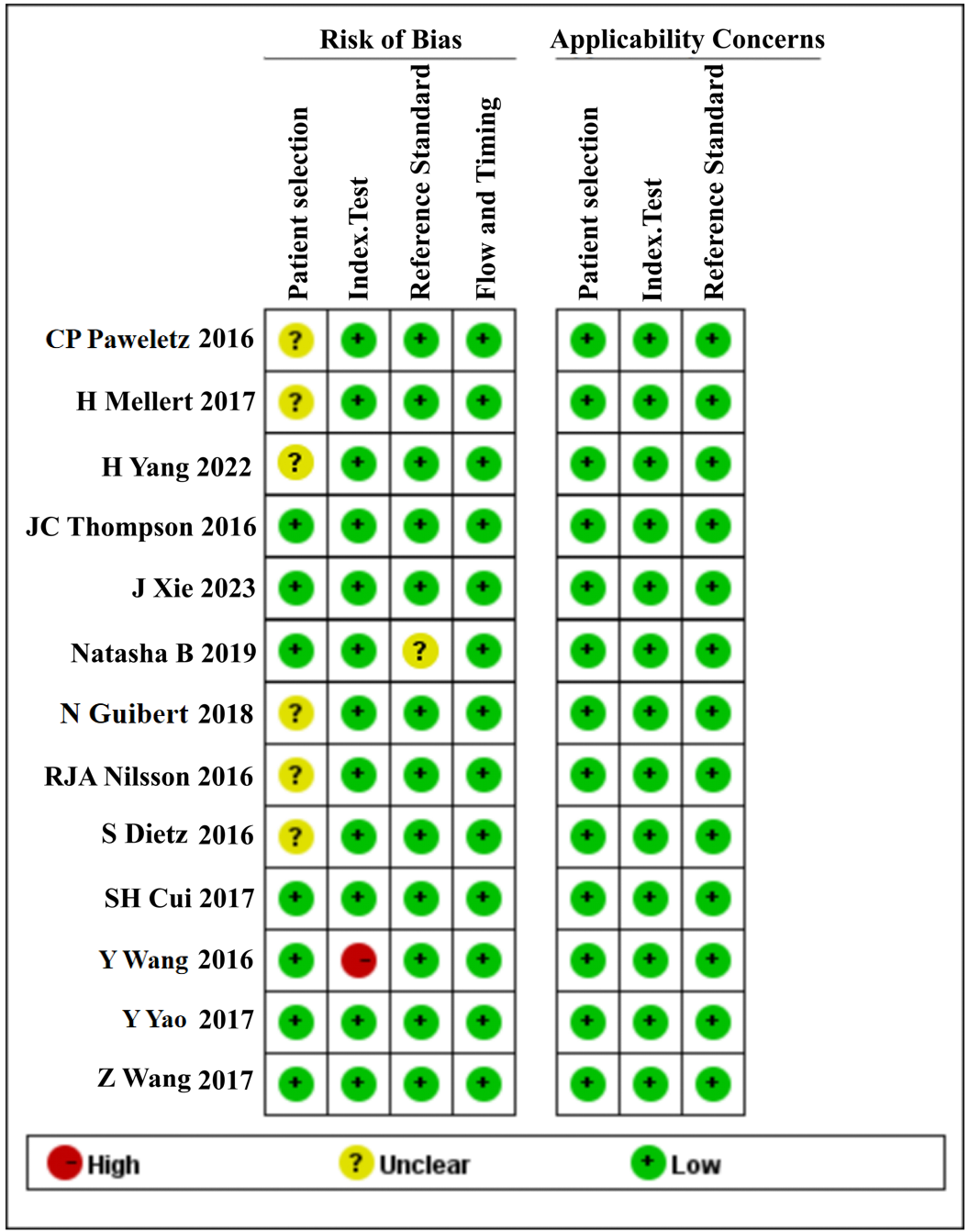

Supplement: S1 Fig — (TIF) [file pone.0330855.s005.tif]

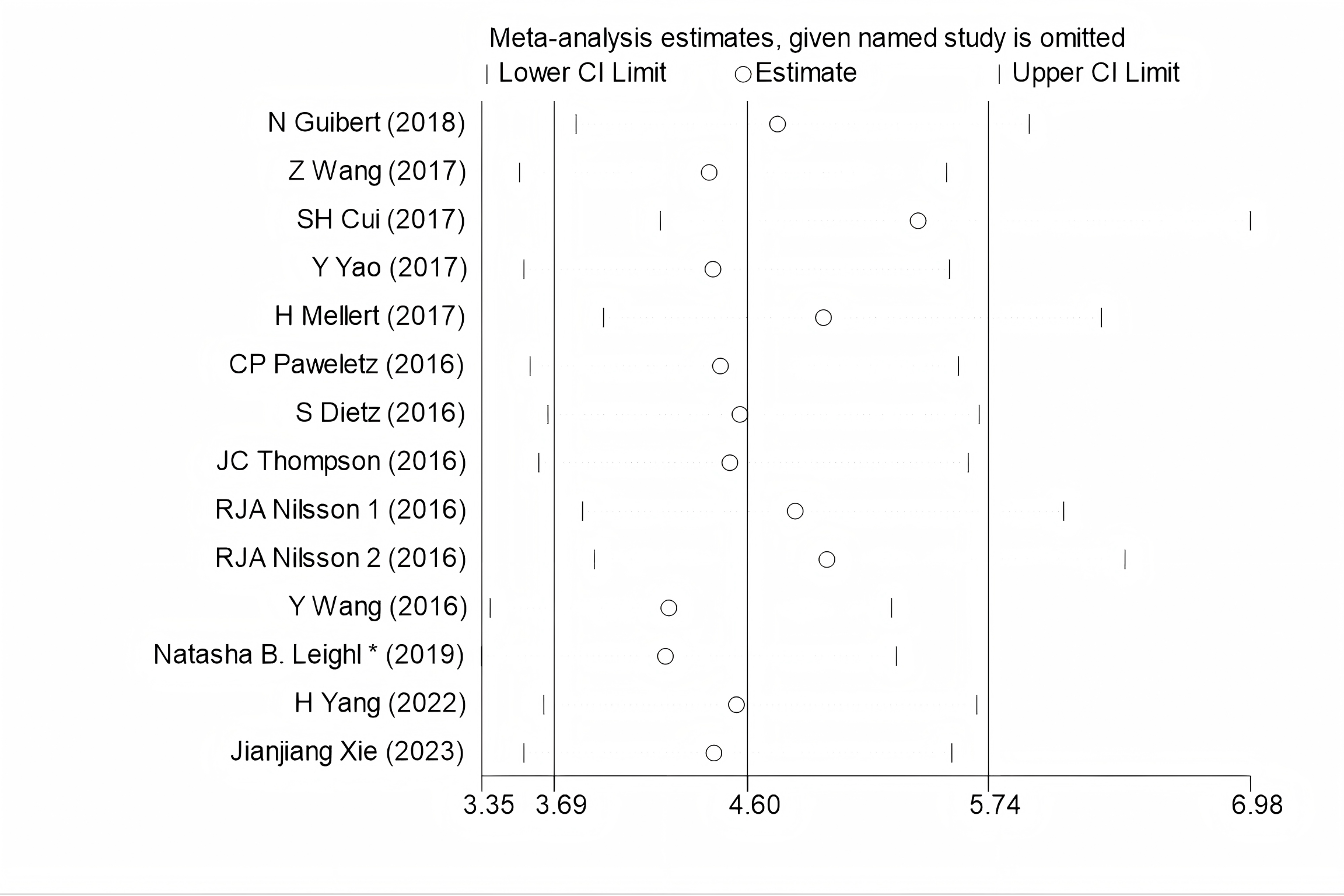

Supplement: S2 Fig — (TIF) [file pone.0330855.s006.tif]

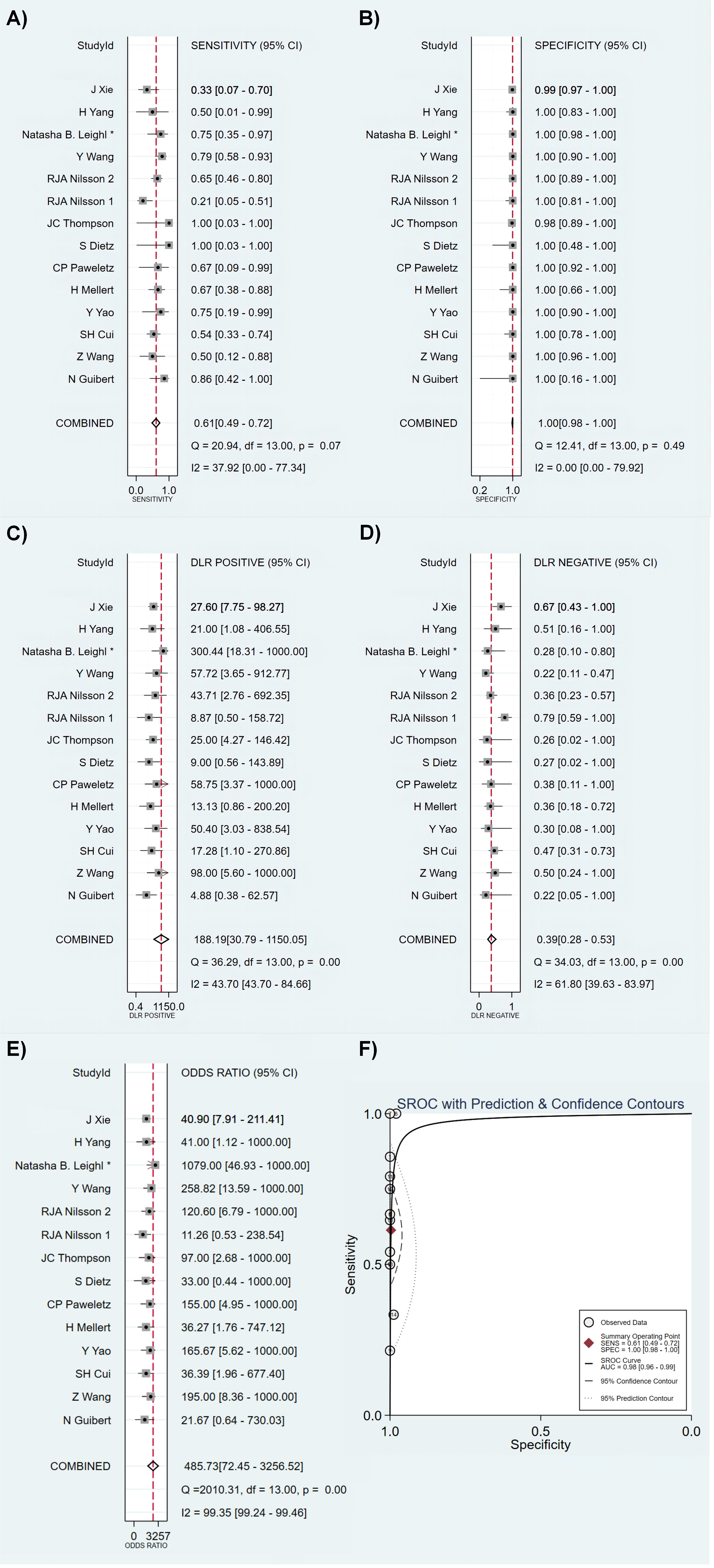

Supplement: S3 Fig — (TIF) [file pone.0330855.s007.tif]
